# Enhancing the Separation Performance of Glassy PPO with the Addition of a Molecular Sieve (ZIF-8): Gas Transport at Various Temperatures

**DOI:** 10.3390/membranes10040056

**Published:** 2020-03-27

**Authors:** Francesco M. Benedetti, Maria Grazia De Angelis, Micaela Degli Esposti, Paola Fabbri, Alice Masili, Alessandro Orsini, Alberto Pettinau

**Affiliations:** 1 Department of Civil, Chemical, Environmental and Materials Engineering, University of Bologna, 40131 Bologna, Italy; micaela.degliesposti@unibo.it (M.D.E.); p.fabbri@unibo.it (P.F.); 2 Department of Chemical Engineering, Massachusetts Institute of Technology, Cambridge, MA 02139, USA; 3 Sotacarbo S.p.A., Grande Miniera di Serbariu, 09013 Carbonia, Italy; alice.masili@sotacarbo.it (A.M.); alessandro.orsini@sotacarbo.it (A.O.); alberto.pettinau@sotacarbo.it (A.P.)

**Keywords:** gas separation, CO_2_ capture, mixed-matrix membranes

## Abstract

In this study, we prepared and characterized composite films formed by amorphous poly(2,6-dimethyl-1,4-phenylene oxide) (PPO) and particles of the size-selective Zeolitic Imidazolate Framework 8 (ZIF-8). The aim was to increase the permselectivity properties of pure PPO using readily available materials to enable the possibility to scale-up the technology developed in this work. The preparation protocol established allowed robust membranes with filler loadings as high as 45 wt% to be obtained. The thermal, morphological, and structural properties of the membranes were analyzed via DSC, SEM, TGA, and densitometry. The gas permeability and diffusivity of He, CO_2_, CH_4_, and N_2_ were measured at 35, 50, and 65 °C. The inclusion of ZIF-8 led to a remarkable increase of the gas permeability for all gases, and to a significant decrease of the activation energy of diffusion and permeation. The permeability increased up to +800% at 45 wt% of filler, reaching values of 621 Barrer for He and 449 for CO_2_ at 35 °C. The ideal size selectivity of the PPO membrane also increased, albeit to a lower extent, and the maximum was reached at a filler loading of 35 wt% (1.5 for He/CO_2_, 18 for CO_2_/N_2_, 17 for CO_2_/CH_4_, 27 for He/N_2_, and 24 for He/CH_4_). The density of the composite materials followed an additive behavior based on the pure values of PPO and ZIF-8, which indicates good adhesion between the two phases. The permeability and He/CO_2_ selectivity increased with temperature, which indicates that applications at higher temperatures than those inspected should be encouraged.

## 1. Introduction

Hydrogen purification was among the first commercial applications that provided potential for large-scale membrane gas separation technologies [1,2,3,4,5]. Polymers entered that market in the 70s due to their low cost, processability, and mechanical properties. However, for polymeric membranes, there is a tradeoff between the permeability, which measures the productivity of the process, and the selectivity, which determines the process efficiency [6,7]. Consequently, there is an upper bound to the performance of polymeric membranes, which makes it difficult to simultaneously enhance the permeability and selectivity. Research on membranes is constantly seeking new materials to improve the membrane performance [8,9,10,11,12]. One way to circumvent the intrinsic limit of the polymers is to combine polymeric materials with selective nanoporous particles. Such fillers can improve the polymer permeability and/or selectivity given their intrinsic superior properties, without compromising those features that make polymeric systems the best choice for industrial applications. The composite membranes thus obtained are usually called mixed-matrix membranes (MMMs). With this aim, many different materials have been dispersed in organic polymers, such as silica particles, zeolites, graphene sheets, carbon molecular sieves (CMS), carbon nanotubes, metal organic frameworks (MOFs), and more recently, covalent organic frameworks (COFs) [13,14,15,16,17,18,19,20,21,22]. In view of all these alternatives, the choice of polymers and fillers that can synergistically combine in MMMs with enhanced properties is of great importance. The use of commercially available materials can address the urgent request to apply membrane technologies on an industrially relevant scale [5,23,24].

Since the discovery of MOFs by Yaghi and co-workers about 15 years ago [25], such materials have attracted the attention of the scientific community because of their exceptional properties and structural tunability, which mean that they can be employed in a virtually infinite range of design. In particular, they have been revealed to be great materials for gas storage and separation applications [24,26]. The first mixed-matrix membrane containing an MOF (i.e., Cu BPDC-TED/PAET) was reported in 2004, and it was tested via single gas permeation measurements [27]. A crucial aspect of the fabrication of MMMs is to ensure good adhesion between the two phases, in order to prevent the formation of non-selective voids at the interface, which can cause an undesired loss in selectivity [28,29]. On the other hand, it is essential to avoid interpenetration between the two phases and keep filler porosities available for gas diffusion, in order to fully exploit their separation ability. MOFs have been proven to have a higher affinity with organic polymer matrices with respect to zeolites, given the organic nature of the linkers that connect the metal clusters to one another [30]. Nevertheless, different strategies have been developed to further increase the interfacial compatibility between the components of MOF-based MMMs [31,32,33].

Zeolitic Imidazole Frameworks (ZIFs) belong to a particular class of MOFs that presents an isomorphism with zeolites [34]. However, the completely inorganic aluminum-silicate structure is replaced by imidazole organic linkers coordinated with metal ions to form ordered frameworks. The crystallographic structure of ZIFs provides them with a monomodal pore size distribution, which is a remarkable feature for separating small gas molecules in the Angstrom scale [35,36]. However, pure MOF membranes cannot reach the expected ideal selectivity towards molecules smaller than the pore diameter because the presence of imperfections such as pinholes and cracks is hard to completely avoid [30]. Such a drawback can be prevented by dispersing ZIFs in a polymer matrix, but in this case, the theoretical separation, obtained based on pore dimension considerations, is hard to reach experimentally due to the flexibility of the metal-organic cage, explained by the so-called “breathing” phenomenon [37,38]. Like all MOFs, ZIFs provide a wide range of designs that can be obtained by changing the imidazolate/imidazolate-like linkers and the coordination metal (e.g., zinc(II) or cobalt(II)) [34]. This leads to different topologies (e.g. *sod*, *rho*, *lta*, *gme*, *gis* etc.) and different dimensions of the pores, which range from 0.7 Å in the case of ZIF-61 to 13.1 Å in the case of ZIF-70. ZIF-8, in particular, features a diameter of the pore (i.e., diameter of the largest sphere that can pass through the entrance of the framework) of 3.4 Å, which lies exactly in between the effective diameter of H_2_ (i.e., 2.90 Å) and that of gases like CO_2_, N_2_, and CH_4_ (i.e. 3.63, 3.66, and 3.81 Å, respectively) [39]. As a consequence, ZIF-8 turns out to be H_2_-selective in terms of its permeability in comparison to those gases [40,41,42,43,44]. Furthermore, ZIF-8 is commercially available, which means that it is potentially readily available for real applications. The combination of these features led us to choose ZIF-8 as a filler in the development of H_2_-selective mixed-matrix membranes. The polymer chosen as the matrix—poly(2,6-dimethyl-1,4-phenylene oxide) (PPO)—is also commercial and is already used in some industrial gas separation membrane modules. It is a glassy amorphous polymer with a good thermal resistance and high permeability in comparison to the standard materials industrially used for gas separation, such as cellulose acetate or polysulfone [45].

The aim of the study was to develop thermally-resistant, H_2_-selective materials to be used in the purification of syngas in processes such as the Integrated Gasification Combined Cycle (IGCC). A membrane-based pre-combustion separation step can reduce the energy consumption associated with the compression of CO_2_ and provide a hydrogen-enriched stream ready to be used as fuel for power generation [46,47].

The membrane preparation was optimized to allow the formation of films with up to 45 wt% of filler, which is a remarkably high amount considering that embrittlement and agglomeration formation become harder to prevent at high loadings [16,48]. The permeation and diffusion of He (used as a model for H_2_), N_2,_ CH_4_, and CO_2_ were investigated at 35, 50, and 65 °C to allow for the calculation of the activation energy. The maximum temperature used in this work was not dictated by the resistance of the membranes, which could work at higher temperatures, but by the operative limit of the permeation apparatus. The optimal, maximum operating temperature for an H2-selective membrane in an IGCC process is around 150 °C [46,47]. Ideally, a temperature of 200 °C would allow the value of the low-temperature shift reactor exhaust gas temperature to be matched and optimize the thermal efficiency. The activation energy results obtained in this work are thus of great importance, because they allow for the extrapolation of transport properties at temperatures higher than experimental ones. Furthermore, pure and composite materials were characterized from a morphological point of view by means of SEM analysis. Microscope images allowed the dispersion and adhesion of the MOF to PPO in the composite films to be assessed. The absence of voids was also verified by performing density measurements of the composite films at different loadings, by comparing the extrapolated value of pure ZIF-8 with that of the theoretical crystal [49]. Thermal properties were investigated by means of TGA and DSC techniques.

## 2. Materials and Methods

### 2.1. Materials

Poly(2,6-dimethyl-1,4-phenylene oxide) was purchased in powder form from *Sigma Aldrich* (*St. Louis, MO, USA)*, and used as received. It is commonly indicated as poly(phenylene oxide) or PPO, and it is a commercial aromatic glassy polymer. In the literature, it has also been referred to by other acronyms, such as PDMPO and PMPO [50,51]. Some of the relevant physical properties of this material are summarized in Table 1.

**Figure 1:**
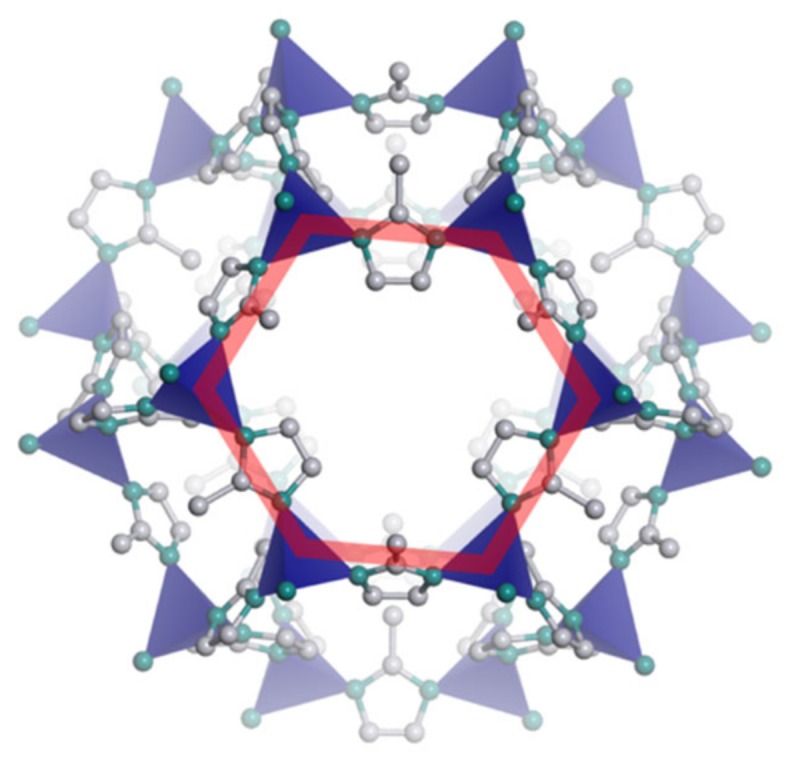
Structure of the Zeolitic Imidazolate Framework 8 (ZIF-8) framework.

The commercial sieve selected to produce the MMMs was ZIF-8 (*Basolite^®^ Z1200, Cat. 691348* produced by *BASF*). In Figure 1, the structure of ZIF-8 is shown. Table 2 summarizes the physical properties, structure, and composition of ZIF-8. The company provided particle size information for ZIF-8, which had a D50 of 4.90 µm. No further grinding was performed on the fillers used to produce MMMs, because the results showed that only small decrements of the particle size could be obtained after the milling process, which is quite invasive and might break the crystalline structure of the materials. Figure 1 highlights the six-membered ring in red, in which the six ZnN_4_ tetrahedra, represented in blue, are connected to one another through organic linkers of 2-methylimidazolate. Carbon atoms are shown in grey, nitrogen atoms are shown in green, and hydrogen atoms are not represented.

ZIF-8 presents a regular zeolite-like sodalite (*sod*) structure. Despite their similar topology, zeolites and ZIFs have a very different chemistry. Zeolites are aluminum-silicate, and thus fully inorganic materials [54]. Conversely, ZIF-8 is a hybrid organic-inorganic material in which metal cations (i.e., Zn^+^) are linked by organic molecules (i.e., 2-methylimidazolate) to form a crystalline and regular structure [30,34,40,49]. ZIF-8 is easier embed in organic polymers, compared with purely inorganic materials, due to the organic part. The vast and recent studies regarding MMMs conducted using ZIF-8 as a filler present evidence for the previous statement [48,55,56,57,58,59,60,61,62,63]. Applications at an industrial scale require materials capable of resisting harsh operative conditions, which can preserve their initial properties over time.

Park et al. [49] investigated the thermal and chemical stability of zeolitic imidazole frameworks, focusing on ZIF-8 due to its exceptional properties. ZIF-8 was demonstrated to possess a high hydrothermal stability, maintaining its architecture, as shown by the PXRD analysis, and its porosity (i.e., sorption capacity) after being exposed to 550 °C in N_2_ atmosphere and after being boiled in water for 7 days. This exceptional result was attributed to the hydrophobicity of ZIFs, which can repel water molecules and avoid attacks of ZnN_4_ units, which can otherwise jeopardize the framework integrity [49]. Küsgens at al. [64] also reported the water sorption isotherm of ZIF-8, which resulted in a negligible amount of water being adsorbed up to $p_{w}/p_{w}^{0}$ = 0.6, where $p_{w}$ is the actual partial pressure and $p_{w}^{0}$ is the saturation partial pressure of water vapor. The behavior was also successfully modeled by using a molecular simulation [65]. Finally, the high Brunauer, Emmett, and Taller (BET) surface area of ZIF-8 of 1630 m^2^/g allows the MOF to have a high sorption capacity [49].

### 2.2. Experimental Methods

#### 2.2.1. Mixed-Matrix Membrane Preparation

The production of homogeneous and stable membranes with a sufficient mechanical resistance, and the development of a simple and reproducible protocol, were crucial steps in this study. It was necessary to optimize several factors, such as the size of the filler particles; the solvent; the solution casting temperature, which directly affects the evaporation rate during casting and the state of the polymer; and finally, the thermal annealing treatment conditions.

##### Membrane Casting

Self-standing PPO and MMM films were obtained through the solution casting technique. A target thickness ranging from 80 to 120 µm was selected. Chloroform (CHCl_3_), 1,1,2-trichloroethylene (TCE), and toluene (TOL) were widely tested in this work under different conditions for both polymeric membranes and mixed matrices, since it has been shown that the choice of the solvent affects the performance of the membranes in terms of gas separation [66,67]. Studies on PPO membrane formation have shown that a decrease in the boiling point (BP) of the solvent leads to a decrease of the permeability and an increase of the selectivity [66]. In addition, PPO may crystallize if the evaporation occurs too slowly, so a more volatile solvent ensures the formation of fully amorphous membranes [66]. Finally, chloroform (*Sigma Aldrich*, purity ≥ 99.8%, *St. Louis, MO, USA*) was selected as the optimal solvent, according to the criteria previously mentioned. Therefore, a similar methodology to the one developed by Aguilar-Vega and Paul [50] was applied to PPO and extended to the MMMs.

The preparation of each solution in this work began by dissolving a 5% by weight of PPO in CHCl_3_. The use of a concentrated solution significantly reduced the sedimentation and the agglomeration of ZIF-8 during the casting step. This was because the high viscosity of the suspension sufficiently reduced the particle mobility, as suggested by Das et al. [68]. Complete dissolution of the polymer in the solvent was reached through magnetic stirring at room temperature for at least 2 h. This solution may be used as such to prepare pure PPO membranes, and as a precursor, in which different quantities of ZIF-8 can be added to obtain MMMs in various percentages by weight of filler in PPO. Prior to use, ZIF-8 was activated at 200 °C under vacuum overnight. At the end of the thermal treatment, the powder was promptly mixed with the polymeric solution under stirring. When the suspension reached a homogeneous condition, it was further sonicated for 4 h (*Lavo, Ultrasonic Vibrator ST-3*). The suspension was poured onto a Petri dish with a diameter of 10 cm, in order to avoid any edge effect on the center of the membrane. The dish was heated at 50 °C and kept on a hot plate to induce quick evaporation of the solvent, which was necessary for obtaining a defect-free material with a good filler dispersion and ensuring the formation of fully amorphous films.

##### Thermal Annealing

Thermal treatment at 150, 200, and 250 °C (with the latter being above $T_{g}$, i.e., 213 °C) was applied to the films to remove the residual solvent and stabilize the gas transport properties over time. A high temperature accelerates the kinetics of the aging process, which would otherwise play a more significant role, although the membranes produced were rather thick (i.e., 80–120 µm), and thus subject to a slower physical aging process.

#### 2.2.2. Density Measurement

Determination of the density of the membranes, $\rho_{MEM}$, was performed by means of the buoyancy method, based on the Archimedes’ principle, using a density kit (*MS-DNY-54*) on a high precision balance (*Mettler Toledo, NewClassic MF MS105DU, Columbus, OH, USA*). Deionized water (*Culligan, M1 Series Commercial Reverse Osmosis Water System*) was used to determine the hydrostatic weight of the sample. A wetting agent (*Pervitro 75% 72409*, included in the density kit) was used to avoid the formation of air bubbles on the submerged film, which might have affected the measurements, introducing a negligible change in the water density. The temperature of the fluid was monitored with a thermometer (± 0.1 °C) to determine the proper density, $\rho_{H_{2}O}$, taken from Perry’s tables [69], in order to calculate the sample density as follows:

(1) $$\rho_{MEM}=\frac{m_{MEM}^{Air}}{\left(m_{MEM}^{Air}-m_{MEM}^{H_{2}O}\right)}\rho_{H_{2}O}\left(T\right)$$

where $m_{MEM}^{Air}$ is the weight of the sample measured in air, while $m_{MEM}^{H_{2}O}$ is the weight measured when the sample was soaked in water. Accurate density values are essential for investigating the volumetric behavior of polymer-filler mixtures and estimating their mixing volume. In particular, the presence of voids inside MMMs would cause a lower-than-additive value of density.

#### 2.2.3. Morphological Characterization

Gas transport properties in composite membranes significantly depend on the filler distribution within the matrix and the adhesion between the particles and polymer. To evaluate these morphological features, field-emission gun-scanning electron microscopy (FEG–SEM) was used (*Fei Company – Bruker Corporation*, *Nova NanoSEM 450, Hillsboro, OR, USA*). All the film samples were fractured in liquid nitrogen, in order to generate a brittle fracture on the cross section to be analyzed. The samples were gold sputtered (*Emitech K550*).

#### 2.2.4. Thermal and Calorimetric Properties

Differential scanning calorimetry (DSC) (*Q20, TA Instrument, New Castle, DE, USA*) was used to optimize the annealing procedure; assess the state of PPO; and evaluate the presence of residual solvent and humidity in PPO, ZIF-8 powder, and MMMs. Thermal transitions were recorded by heating samples under N_2_ flux at a 10 °C/min rate, from 25 to 300 °C. Two heating scans were recorded for each sample.

Thermal gravimetric analysis (*Q50, TA Instrument, New Castle, DE, USA*) experiments were performed to investigate the thermal stability of pristine materials and MMMs. Samples of about 20 mg were held in a platinum pan and were heated to 200 °C in N_2_ atmosphere and left for 1 h in these conditions, in order to make sure that all residual water and gases were removed. After that, the temperature was increased to 800 °C in the same atmosphere at a constant rate of 10 °C/min.

#### 2.2.5. Gas Permeability Experiments

**Figure 2:**
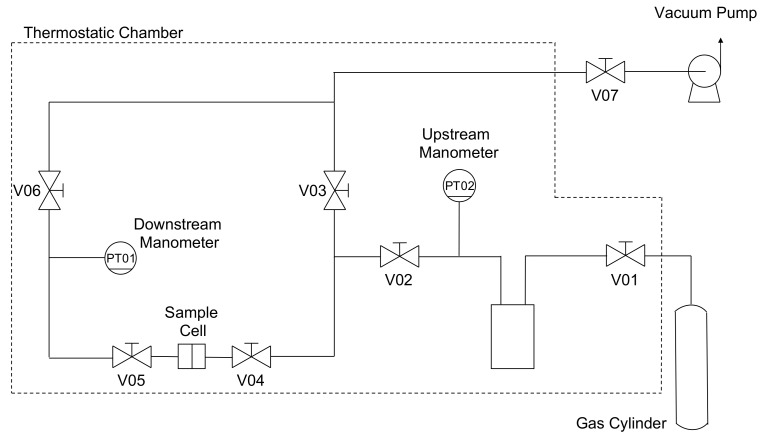
Layout of the permeation equipment. The outer black dashed line indicates the volume in which temperature is controlled.

The permeability of He, N_2_, CH_4_, and CO_2_ was evaluated at different temperatures (i.e., 35, 50, and 65 °C) for pure PPO and MMMs at different loadings of the filler up to 45 wt% of ZIF-8. All gases were purchased from *S.I.A.D. Spa* (Bergamo, Italy) with a purity of or above 99.99% and used as received. The order in which gases were tested was as follows: He, N_2_, CH_4_, and CO_2_. This was pursued to prevent any conditioning effect of the sample, as PPO undergoes plasticization when exposed to high pressures of CO_2_ [70,71,72]. Each permeability experiment was performed at an absolute upstream pressure of 1.3 bar; thus, the pressure was low enough to prevent the plasticization effect. However, to make sure that the membrane permeability was unchanged after tests with CO_2_, the helium permeability was measured again at the same conditions. To investigate the effect of the temperature at different loadings of the sieve, the permeability of the four gases was evaluated at 35 °C, 50 °C, and eventually 65 °C for the same sample, avoiding any difference due to the change of the membrane. The fixed-volume variable-pressure manometric technique previously described elsewhere [73] was implemented to perform the experiments. The essential layout of the equipment is shown in Figure 2.

A circular self-standing film was placed in the sample holder, which was a stainless-steel cell, and sealed by means of an O-ring made of Viton^®^ to ensure that the system was leak-tight. A forced ventilation thermostatic chamber (*Type M 150-TBR, MPM Instruments S.r.l., Bernareggio, Italy*) was used to control the air temperature with an accuracy of ±0.1 °C. The specimen was conditioned under dynamic vacuum overnight to remove any possible species from the matrix, such as gases and humidity coming from the brief exposition to air. Once equilibrium conditions were achieved, V04 was opened to start the experiment. The increase of the downstream pressure in the calibrated closed volume, $V_{d}$, was monitored by a capacitance manometer (PT01 - *Barocel^®^ Edwards, Burgess Hill, UK*) with a sensitivity of 10^−2^ mbar and an accuracy of 0.15% of the reading. Since the initial downstream pressure, $p_{d}$, was a vacuum, the permeability could be evaluated at the steady state by means of the following equation:

(2) $$P=\frac{V_{d}}{RT}\frac{l}{A}\frac{1}{\left(p_{u}-{\bar{p}}_{d}\right)}\left[{\left(\frac{dp_{d}}{dt}\right)}_{SS}-{\left(\frac{dp_{d}}{dt}\right)}_{leak}\right]$$

in which R is the gas constant, T is the operative temperature, l is the membrane thickness, and ${\bar{p}}_{d}$ is the average downstream pressure of the considered gas. ${\left(\frac{dp_{d}}{dt}\right)}_{SS}$ and ${\left(\frac{dp_{d}}{dt}\right)}_{leak}$ are the changes in pressure at a steady state (SS) and when the equipment was sealed under static vacuum (leak). The uncertainty of the permeability values was calculated by considering the experimental error made to measure l, ${\bar{p}}_{d},$ and $V_{d}$, by means of the propagation of error approach [74]. The ideal selectivity between gas A and B, $\alpha_{A/B}$, could be calculated for each gas pair as follows:

(3) $$\alpha_{A/B}=\frac{y_{A,d}/y_{B,d}}{y_{A,u}/y_{B,u}}≅\frac{P_{A}}{P_{B}}=\frac{D_{A}}{D_{B}}\frac{S_{A}}{S_{B}}=\alpha_{\frac{A}{B}}^{D}\alpha_{\frac{A}{B}}^{S}$$

where $y_{A,d}$ and $y_{B,d}$ are the molar fraction on the downstream side of the membrane of gas A and B, respectively, while $y_{A,u}$ and $y_{B,u}$ are those on the upstream side of the film. $P_{A}$ is the permeability of the more permeable gas of the pair and $P_{B}$ is that of the less permeable one. Furthermore, the ideal selectivity could be split into two contributions: $\alpha_{A/B}^{D}$, which is the diffusivity selectivity, and $\alpha_{A/B}^{S}$, which is the solubility selectivity.

The time-lag, $\theta_{L}$, was evaluated for all the gases by using the time-lag method. The time-lag is a measure of the characteristic time required for the gas molecule to dissolve in the polymer matrix and diffuse through the film. Considering the operative conditions under which the experiments were performed, i.e., a zero initial concentration of gas across the membrane, $\theta_{L}$ can be related to the diffusivity, D, through the following equation [75,76]:

(4) $$D=\frac{l^{2}}{6\theta_{L}}$$

The diffusivity and permeability can be described by an Arrhenius-like equation [58,77], and analogous formulations can be provided as follows:

(5) $$P=P_{\infty }exp\left(-\frac{E_{P}}{RT}\right)$$

(6) $$D=D_{\infty }exp\left(-\frac{E_{D}}{RT}\right)$$

where $E_{D}$ is the activation energy of the diffusion process, which is the barrier that needs to be overcome by a gas molecule to make a diffusive jump from one cavity to another, and $D_{\infty }$ is the temperature-independent pre-exponential term, which represents the diffusion coefficient at an infinite temperature. Similar considerations hold for permeability, with the essential difference that permeation is not a thermally-activated process, since permeability is a combination of a kinetic and thermodynamic factor, and both increasing and decreasing trends can be experienced as a function of temperature. However, the energetic constant of permeation, $E_{P}$, can be calculated by means of (5), and the pre-exponential factor, $P_{\infty }$, as for diffusion, is temperature-independent and represents the permeation coefficient at an infinite temperature. Proper fitting provides the possibility to extrapolate experimental permeability and diffusivity values at higher temperatures than those investigated. This is very useful information, since these MMMs might be required to work at a high temperature, with H_2_/CO_2_ separation favored under those conditions and materials compatible with such a high temperature. Eventually, the heat of sorption, $\Delta H_{S}$, of each gas in the MMMs can be calculated by simply subtracting the two contributions as follows:

(7) $$\Delta H_{S}=E_{P}-E_{D}.$$

## 3. Results and Discussion

### 3.1. Aspect of the MMMs

**Figure 3:**
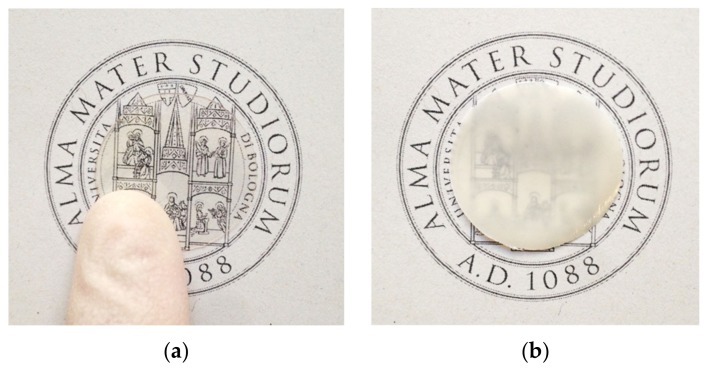
Membrane samples used for permeation tests. (**a**) PPO and (**b**) 25 wt% ZIF-8/PPO.

The PPO-based MMMs at different loadings of ZIF-8 were prepared following the optimized protocol described above. In Figure 3, it is possible to appreciate the transparency of pure PPO membranes, while MMMs with ZIF-8 developed a slight haze due to the presence of fillers. Composite membranes were found to be macroscopically homogeneous, testifying to the overall good dispersion of the particles inside the polymer matrix.

Membranes made of polymer have a higher flexibility. MMMs with a filler content up to 15 wt% are still robust and preserve this feature. At intermediate loads (e.g., 25 wt%), membranes are less robust. Films begin to become more brittle when the particle content increases up to 45 wt%. These materials revealed a lower resistance to bending, which was expected since, for instance, a ZIF-8/PPO membrane at 45 wt% contains about a 48% volume of the MOF. A detailed and quantitative characterization of the effect of filler on the mechanical properties of the membranes was beyond the scope of this work, which was aimed at preliminarily assessing the effect of filler on the transport properties. For this purpose, it is a requirement that the composite membranes resist the pressure difference applied across the membrane and the stress imposed by leak-proof tightening of the permeation cell, without inducing pinholes or cracks. This requirement was fulfilled by all membranes with up to 45 wt% of filler.

### 3.2. Density

**Figure 4:**
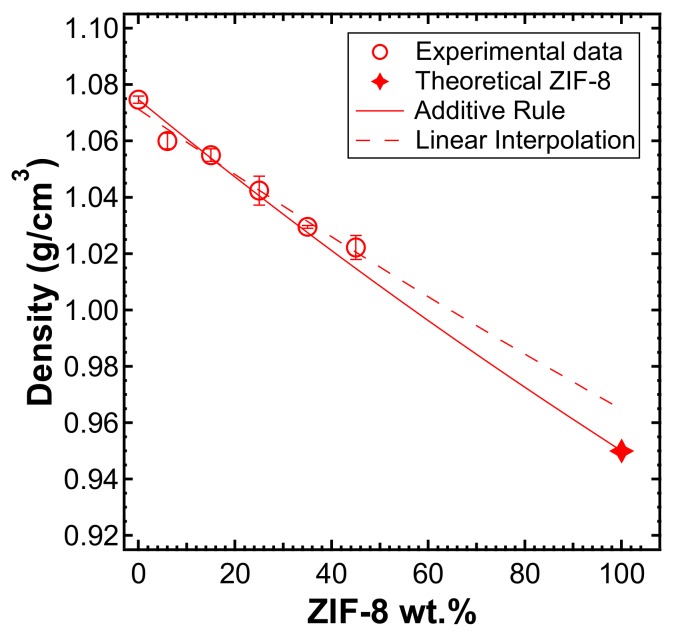
Density of the mixed-matrix membranes (MMMs) versus ZIF-8 weight fraction in the film (empty circles), measured with the buoyancy technique in water. Solid line represents the additive rule and was evaluated by the experimental density of PPO and the theoretical density of ZIF-8. Dashed line represents the linear interpolation of the experimental values measured, extrapolated to pure ZIF-8.

The density values of pure materials and composite membranes versus filler content are reported in Figure 4. The density of the MMMs decreased when increasing ZIF-8 loading. By plotting the same data in terms of the specific volume of each film as a function of weight filler loading, the expected linear correlation (${\hat{\nu }}_{MMM}=w_{PPO}{\hat{\nu }}_{PPO}+w_{ZIF}{\hat{\nu }}_{ZIF}$) was obtained with a correlation coefficient of R^2^ = 0.98 (represented with a dashed line in Figure 4). Hence, it was possible to estimate the density of ZIF-8 by extrapolating the linear function. Surprisingly, the ZIF-8 density was 0.96 g/cm^3^, which is a value very close to that of the theoretical density of the regular ZIF-8 crystal, i.e., 0.93–0.95 g/cm^3^ [49,55,61]. This may indicate that the presence of voids inside the MMMs is negligible, since interfacial adhesion defects would be detected by the lower-than-ideal values of density of the membranes. The additive rule for composite materials is shown in (8):

(8) $$\rho_{MMM}=\frac{\rho_{PPO}\rho_{ZIF}}{w_{PPO}\rho_{ZIF}+w_{ZIF}\rho_{PPO}}$$

and it is represented as a solid red line in Figure 4. The additive rule was implemented to compare the experimental values with the ones predicted by the ideal combination of the two phases. The consistency between the additive rule and the actual density of the composites indicates that the polymer and filler phase display good adhesion. This does not necessarily indicate that the filler phase is evenly distributed in the matrix, but rather that there is no void formation at the polymer/filler interface, which may compromise the intrinsic selectivity of the composite materials.

### 3.3. SEM Analysis

**Figure 5:**
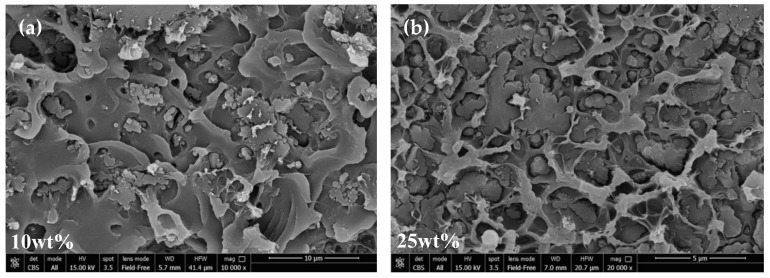

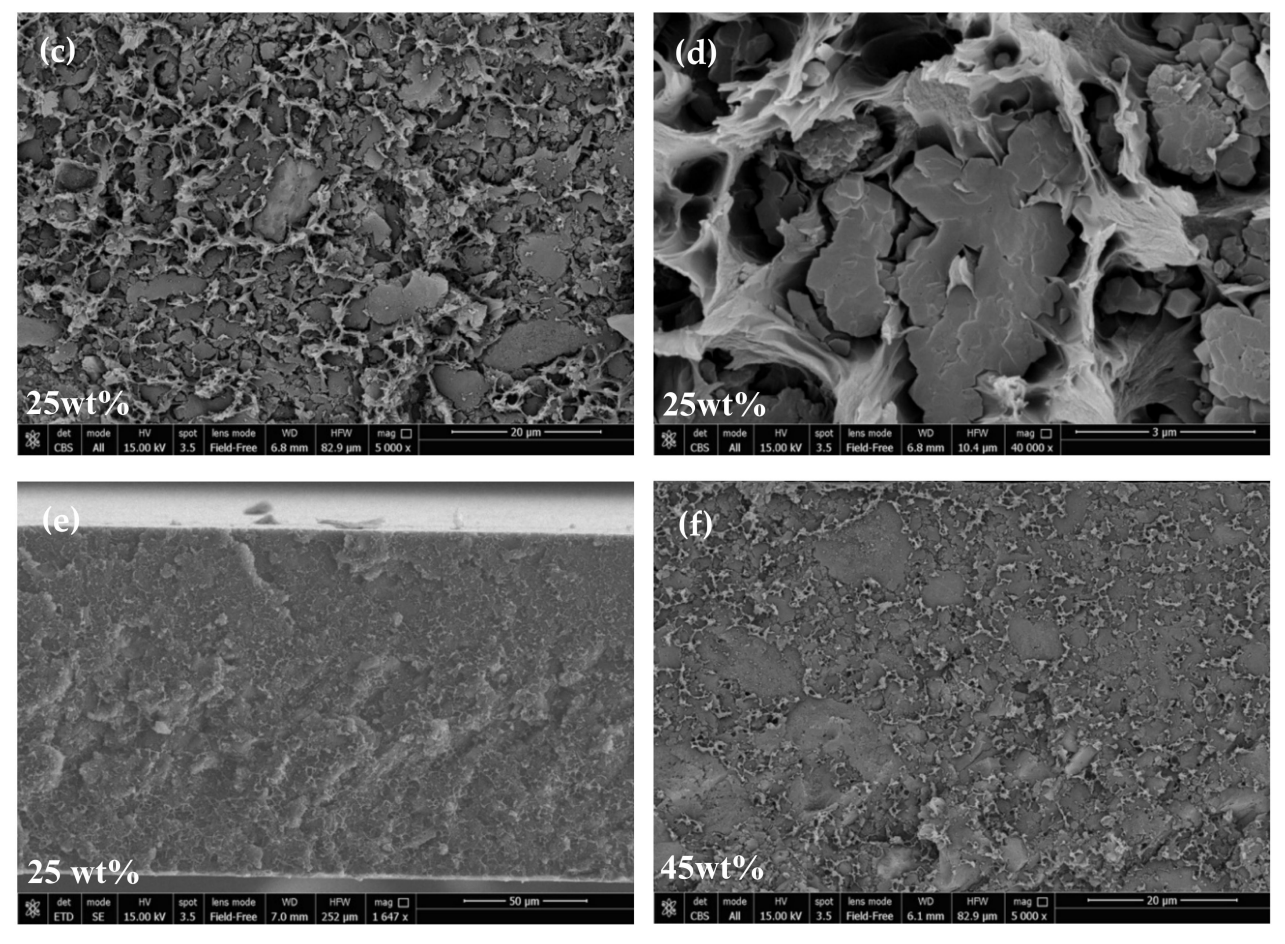
SEM images of the cross-section of ZIF-8/PPO mixed-matrix membranes at different loadings and magnitudes: (**a**) 10 wt%, (**b**–**e**) 25 wt%, and (**f**) 45 wt%. Other images can be found in the Supplementary Information.

The morphology of ZIF-8/PPO MMMs was investigated at different loadings of the MOF by means of SEM analysis. This enabled us to determine the quality of adhesion between the particles and polymer matrix, as well as evaluate the dispersion of the filler. The SEM images are reported in Figure 5, which generally shows that ZIF particles and the polymer are compatible and had good adhesion. This is consistent with the partially organic nature of the filler, which improves the affinity with the polymer matrix, as described above. Additional SEM pictures are reported in the Supplementary Information in Figure S3.

However, detachment at the interphase appears to happen in some cases, and this can be explained by the following. It is common knowledge that glassy polymers with a rigid backbone and a high glass transition temperature, such as PPO, vitrify when the solvent evaporates [18]. The evaporation-induced transition from a rubbery state to glassy state can cause significant stress to the system. As also pointed out by Koros et al. [28], this phenomenon can happen during composite membrane formation before all of the solvent has left the film, making further evaporation beyond this point crucial for detachment of the polymer chain from the filler. This could lead to the formation of non-selective voids, which might prevent the membranes from being as selective as expected, but, on the other hand, make them more permeable. In this work, slight delamination between the two phases can be seen, especially at high loadings (i.e., ≥25 wt%). However, the presence of non-selective voids can be excluded, since no anomalous selectivity loss was observed with increasing filler loading, as will be discussed in the following sections, and the composite density closely followed the volume additivity, as shown above. As pointed out by Ordonez et al. [48], delamination can also be induced by fracturing the membranes with liquid nitrogen prior to SEM analysis. The latter contribution would not affect the transport properties, being caused artificially during the preparation of the sample for the analysis. However, we believe that the mechanical stress imposed to break the films would be responsible for the detachment to some extent. Chung et al. [18] reported the use of a higher-than-ambient temperature to promote fast evaporation during film formation, and in the case reported here, a net heat flux and temperature gradient between the bottom and top of the nascent membrane were generated. This way of heating was found to promote convective fluxes inside the fluid suspension, leading to inhomogeneous thicknesses and irregular distributions of the filler in the resulting MMMs. However, these kinds of consequences were not experienced by the ZIF-8/PPO membranes, probably because of the high viscosity of the casting solution. Overall, the distribution of filler within the matrix was ubiquitous, although in a small number of cases, larger aggregates were visible. The quick solution casting (i.e., 15 min) and the high viscosity of the solution, were not sufficient to completely eradicate this phenomenon. Nevertheless, the majority of particles were smaller (e.g., 1–4 µm) and many ZIF nanocrystal cubes with a side of ≈200 nm could be observed (Figure S3).

### 3.4. DSC Tests

**Figure 6:**
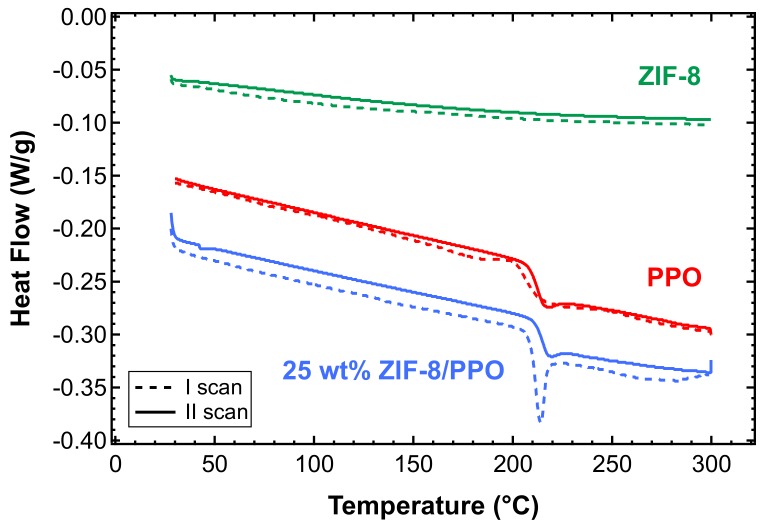
Differential scanning calorimetry (DSC) tests on pure amorphous PPO film after a thermal annealing treatment at 200 °C under vacuum (red), ZIF-8 powder as received (green), and 25 wt% ZIF-8/PPO mixed-matrix film after undergoing a thermal annealing treatment at 200 °C under vacuum (blue).

DSC analysis was carried out in two subsequent runs on films of PPO, ZIF-8 powder as received, and mixed-matrix films, between 25 and 300 °C. Figure 6 shows that the pure PPO films obtained via solvent casting in chloroform at 50 °C were fully amorphous, as they showed the typical glassy transition peak at about 213 °C, consistent with literature values, in both scans [50,53]. It must be noted, however, that slower casting at room temperature (i.e., complete evaporation in 3 days) results in the formation of semi-crystalline PPO samples, as verified via DSC analysis (Figure S2a), and rupture of the membrane during film formation (Figure S2b). A specific analysis was carried out in this work on the effect of the casting temperature on the final properties, although it is not reported here because it is beyond the scope of the paper. The value of 50 °C appears to be the optimal one for producing robust and amorphous PPO films. There is almost no difference between the first and second scan in the amorphous PPO, because the sample was previously treated at 200 °C under vacuum, proving that the treatment can remove any residual solvent.

Almost no difference was observed between the two scans carried out on ZIF-8, which indicates that the material does not retain a significant amount of water due to its hydrophobicity, as indicated in the literature (Figure 6) [49,64,65]. The observation comes from the fact that hydrophilic materials, such as zeolites, show broad endothermic peaks in DSC scans performed in the same range of temperatures, as well as when specimens are stored in environmental conditions prior to the test [78].

The same analysis was carried out on MMMs containing PPO and different amounts of ZIF-8. Figure 6 shows the results relative to the sample containing 25 wt% of ZIF-8. One can notice a sharper peak upon transition in the first scan, which indicates enthalpic relaxation at $T_{g}$. Such a sharp peak is absent in the second scan.

**Figure 7:**
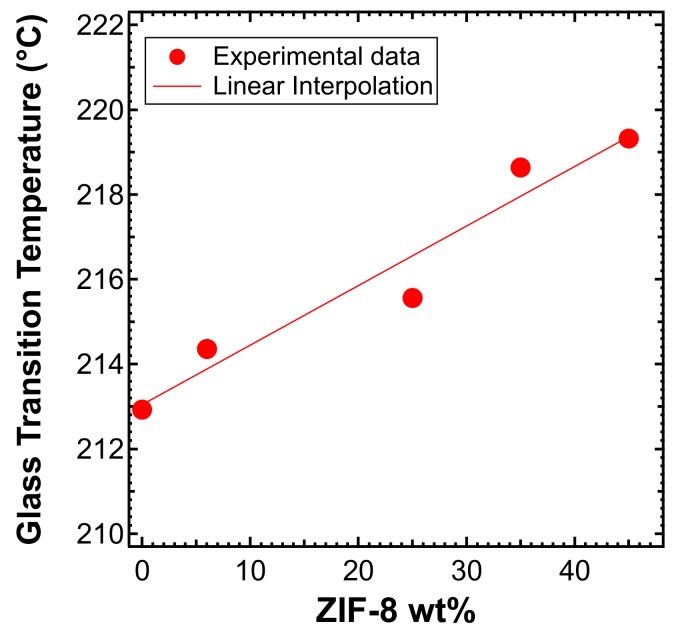
Trend of the glass transition temperature in ZIF-8/PPO MMMs as a function of the filler loading.

The addition of an increasing amount of ZIF-8 increases, albeit slightly, the glass transition temperature of the PPO, as shown in Figure 7. Previous works that tried to correlate the variation of permeability and selectivity with the variation of $T_{g}$ of MMMs did not provide clear and univocal conclusions. In particular, Moaddeb et al. [77] observed that the reduction in the mobility of 6FDA-IPDA chains conferred an enhanced O_2_/N_2_ selectivity, while the permeability was barely altered or negligibly reduced. The opposite was observed by Song et al. [55]. In their work, ZIF-8/Matrimid^®^ membranes showed an enhanced permeability up to about 3–4 times that of pure Matrimid^®^, while the H_2_/CO_2_ selectivity remained constant with the filler loading (i.e., with increasing $T_{g}$). These results are in contrast to what was reported by Díaz et al. for ZIF-8/PPEES systems, where no changes of $T_{g}$ were observed [60]. In the case of the composite materials studied in the present work, we believe that the slight increase of $T_{g}$ should not affect the gas transport properties in the polymer phase, because at temperatures far below $T_{g}$ and pressures far below the plasticization point, the different rigidity of the various matrices should not play a role.

### 3.5. Permeability and Permselectivity

#### 3.5.1. Gas Transport in PPO and ZIF-8

PPO has been widely studied in the framework of gas separation because of its excellent sorption and transport properties [50,52,79,80]. As mentioned by Toi et al. [80], PPO shows a higher permeability and sorption than other commercial glassy polymers with a rigid chain backbone. The high extent of sorption can be ascribed to the high glass transition temperature ($T_{g}\approx 213\;{}^{\circ}C$) [50,52,80], which indicates a high amount of non-equilibrium excess-free volume. The relatively high permeability is related to the high diffusion coefficients of low-weight penetrants, which stem from the high Fractional Free Volume (FFV) of the polymer, which was measured to be about 19% by Huang and Paul [52]. Along with these properties, other features that make PPO suitable for industrial applications are its relatively low cost, compared to other common techno-polymers, and the possibility to work with it at high temperature [53]. This is an important aspect when the separation process is controlled by the diffusivity of the gas species in the membrane, as in this case. Despite gas permeability values showing discrepancies in the literature, different studies have revealed that PPO is highly permeable to H_2_ (i.e., 86.9–112.8 Barrer), instead demonstrating a moderate selectivity for the H_2_/CO_2_ couple at room temperature (i.e., ideal perm-selectivity range between 1.49 and 1.54) [51,81,82]. PPO, in particular, behaves as a molecular sieve, and permeability values can be arranged in the following order: $P_{H_{2}}>P_{He}>P_{CO_{2}}>P_{N_{2}}≅P_{CH_{4}}$, which is almost the opposite trend of the kinetic diameter, for which the order is $d_{He}<d_{H_{2}}<d_{CO_{2}}<d_{N_{2}}<d_{CH_{4}}$ [50,51]. It is worth stressing that hydrogen is the most permeable gas in PPO, which makes the use of helium as a model for hydrogen, in this work, a conservative choice for estimating the performance of H_2_/CO_2_ separation.

ZIFs, such as ZIF-7 and ZIF-8, and zeolites, such as Zeolite 3A, have pore sizes that approach the size of gas molecules, which is a feature that makes them theoretically capable of performing gas separation with a very high selectivity towards smaller gases. In particular, for ZIF-8, the diameter of the apertures is estimated to be 3.4 Å based on crystallographic data, and is thus larger than H_2_’s effective diameter (2.90 Å), but smaller than that of CO_2_, N_2_, and CH_4_ (3.63 Å, 3.66 Å, and 3.81 Å, respectively) [39]. Bux et al. [40,42], as well as McCarthy et al. [41], reported that ZIF-8 is an H_2_-selective material, as far as H_2_/CO_2_ separation is concerned, having performed experiments with both pure and mixed gases. The measurements showed that there is no sharp cut-off between molecules smaller and bigger than the pores. The fact that molecules with a kinetic diameter larger than the pores can permeate through metal-organic networks was studied in detail by Caro [30]. He found that MOFs often exhibit a pronounced structural flexibility, which makes the framework of these materials less rigid than that of zeolites. Furthermore, Bux and coworkers [40] noticed that the H_2_ flux in ZIF-8 membranes in the presence of co-permeating CH_4_ is only slightly affected by the presence of the larger molecule in the mixture, and the permeation results are comparable to those of single-gas permeability. This behavior was ascribed to the fact that, even though the pore size of ZIF-8 is small, the space inside the largest cage of the system is far larger, thus accommodating a sphere with a diameter as big as 11.6 Å. The values of d_a_ (diameter of the aperture by which molecules can enter the framework) and d_p_ (diameter of the largest sphere that can fit into the largest cavity of the crystalline structure) are reported in Table 2 [34]. Therefore, once CH_4_ enters the cage and frees the 3.4 Å-wide pore of ZIF-8, H_2_ can diffuse through the network. The organic nature of the filler makes the framework flexible, causing values of selectivity that are lower than expected. However, its organic nature is essential for having a good compatibility with the polymer [37,38]. The permeability across a pure ZIF-8 membrane has been measured by various authors and the results are reported in Table 3. The H_2_ permeability ranges between 4916 and 10,333 Barrer. However, the selectivity values are moderate, possibly due to the flexible morphology of this material. Therefore, a dramatic increase of the membrane selectivity, at least at low and moderate temperatures, is not to be expected upon the addition of ZIF-8.

#### 3.5.2. Thermal Annealing

Physical aging is a phenomenon that occurs in all amorphous materials in a glassy state which evolve towards an equilibrium point and is accelerated by high temperatures. Indeed, it was shown by Ansaloni et al. [83], who performed studies on another glassy polymer, Matrimid^®^ polyimide, that the increase of the thermal treatment temperature led to a larger reduction of the FFV and a stabilization of transport properties over time. Savoca et al. [84] observed a considerable decrease of permeability in poly(1-trimethylsilyl-1-propyne) (PTMSP) films when increasing the temperature of the thermal treatment, testifying that the sample returned to its original permeability after dissolving and recasting the membrane. Hung and Paul’s studies have demonstrated that the aging rate of PPO is faster the higher the aging temperature, and slower the thicker the membrane, by monitoring key parameters, such as the FFV, gas permeability, and refractive index of the polymer [52,85,86]. At 35 °C, a PPO film with a thickness of ≈400 nm, experienced a loss of permeability of ≈65% over 4000 h (≈6 months); conversely, a ≈25 µm film revealed a decrease of ≈20% over 10000 h (≈14 months) [85].

**Figure 8:**
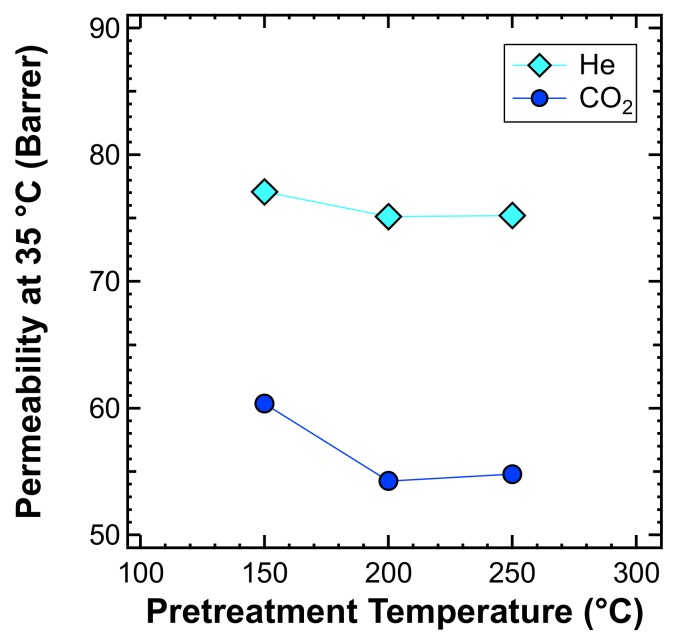
Effect of the thermal annealing temperature on the pure PPO permeability at 35 °C.

For these reasons, whenever starting a comprehensive experimental campaign on a glassy membrane system, it is necessary to stabilize the membrane properties. We carried out a specific study to locate the minimum annealing temperature required to reach a stable permeability and solvent-free films. The study was carried out by measuring the permeability of He and CO_2_, at 35 °C, after thermal treatment carried out under vacuum at various temperatures, from 150 to 250 °C, overnight. The results of gas permeability plotted against the pretreatment temperature are reported in Figure 8**.** It shows that the permeability decreased when increasing the treatment temperature, due to the accelerated aging induced by such high temperatures, and reached a plateau at a temperature of 200 °C, which is also below the $T_{g}$ of PPO. Therefore, such tests allowed us to identify the optimal treatment temperature as being equal to 200 °C.

It must also be noted that high-temperature treatment can replace a stability test on the membrane; indeed it induces accelerated ageing, which decreases the permeability of the membranes by quickly compacting and densifying the polymer chains. Such treatment thus reduces the need to perform a time-consuming lifetime test that will require the membrane to be naturally ageing across periods of months or even years. Filled membranes have similar responses to thermal treatment than unfilled ones, so the addition of filler does not seem to impact the expected durability of the material.

#### 3.5.3. Effect of the Filler Loading

**Figure 9:**
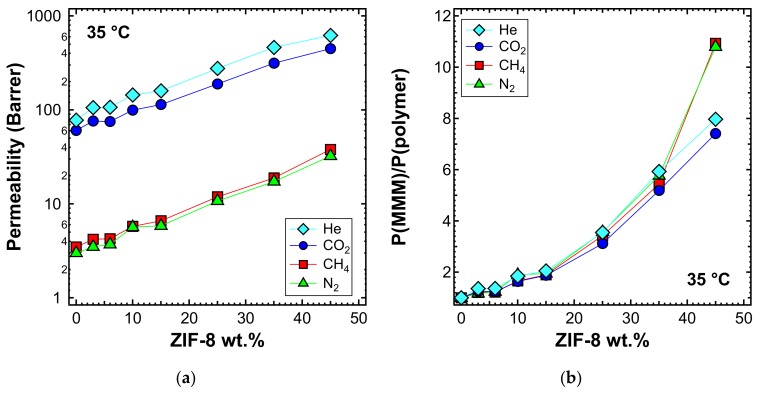
(**a**) Permeability and (**b**) relative permeability increase of various gases at 35 °C with an upstream pressure of 1.3 bar in ZIF-8/PPO MMMs.

Pure gas permeability tests with He, N_2_, CH_4_, and CO_2_ were performed for several membranes at 35 °C, covering the whole range of filler loadings investigated, namely 0%, 3%, 6%, 10%, 15%, 25%, 35%, and 45% by weight. Figure 9 shows that the permeability increases monotonously with the filler loading. The permeability enhancement is extremely high and, in particular, adding 45 wt% of ZIF-8 to PPO enhances the He permeability by a factor of about 8. Table 4 presents the ideal selectivity for relevant gas pairs (i.e., He/CO_2_, CO_2_/N_2_, CO_2_/CH_4_, He/CH_4_, and He/N_2_), evaluated by means of Equation (3). The uncertainty of the calculated permeability primarily originated from the variation of membrane thickness. However, the overall variability was always kept within ±3% for MMMs up to 25 wt% and within ±8% for higher loadings.

**Figure 10:**
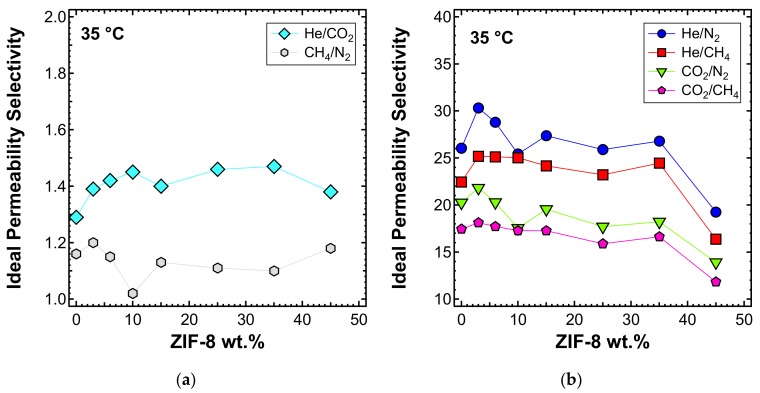
Ideal selectivity for (**a**) He/CO_2_ and (**b**) other gas couples at 35 °C with an upstream pressure of 1.3 bar in ZIF-8/PPO MMMs.

The significantly enhanced permeability was accompanied by a modest increase in selectivity for the He/CO_2_ gas pair (i.e., up to 15% more than that of pure PPO). Similar results were achieved by other authors [55,56,60,87]. The MMM permeability results are in line with what was expected for the transport properties of pure PPO measured in this work, and ZIF-8 permeability and ideal selectivity data from the published literature summarized in Table 3. In fact, the remarkable enhancement of permeability can be attributed to the very high permeability of ZIF-8, which is about two orders of magnitude higher than that of pure PPO. This result demonstrates that the filler actively contributes to the transport of gas molecules and that its pores are not blocked by the polymer phase. Furthermore, ZIF-8 shows a higher H_2_/CO_2_ ideal selectivity than PPO, and this also led to a small improvement of He/CO_2_ selectivity in the ZIF-8/PPO composite membranes up to 35 wt% (Figure 10a). Conversely, CO_2_/N_2_, CO_2_/CH_4_, He/CH_4_, and He/ZIF-8 ideal selectivity for ZIF-8 was lower than that of PPO, so a slight decreasing selectivity was expected by MMMs. At loadings up to 35 wt%, the results matched the expectations, while when the 45 wt% loading was reached, a more pronounced decrease was measured (Figure 10b), likely due to the formation of a small amount of non-selective voids. This was further confirmed by the anomalous permeability increase for N_2_ and CH_4_—the bigger gas molecules—presented in Figure 9b.

**Figure 11:**
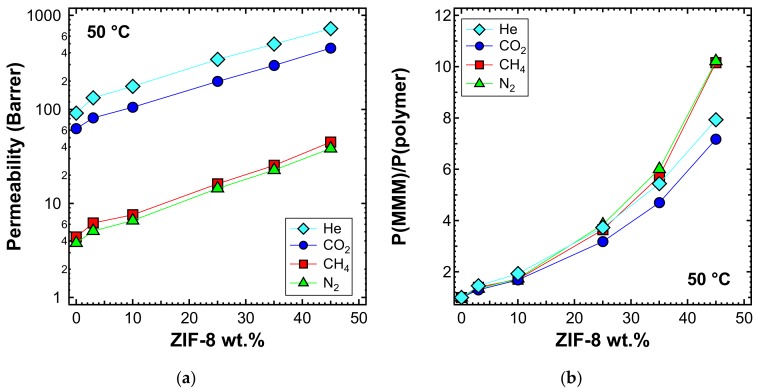
(**a**) Permeability and (**b**) relative permeability increase of various gases at 50 °C with an upstream pressure of 1.3 bar in ZIF-8/PPO MMMs.

**Figure 12:**
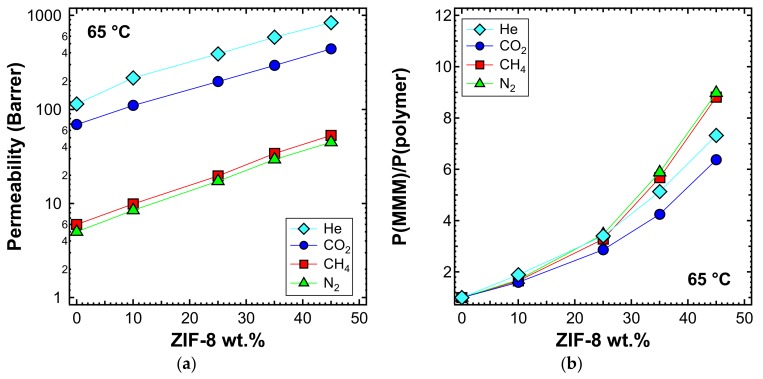
(**a**) Permeability and (**b**) relative permeability increase of various gases at 65 °C with an upstream pressure of 1.3 bar in ZIF-8/PPO MMMs.

Permeability tests were also performed at 50 and 65 °C for a selected list of MMM samples. When viewing the behavior at different temperatures, one can notice that the qualitative trends were similar, albeit with generally higher values of permeability, which are shown in Figure 11a (50 °C) and Figure 12a (65 °C). The relative increase of permeability, which is displayed in Figure 11b (50 °C) and Figure 12b (65 °C), was slightly lower than what was observed at 35 °C in Figure 9a. In particular, we noticed that at higher temperatures, the effect of adding ZIF-8 on the permeability is around the same for He and CO_2_, while less marked for N_2_ and CH_4_.

**Figure 13:**
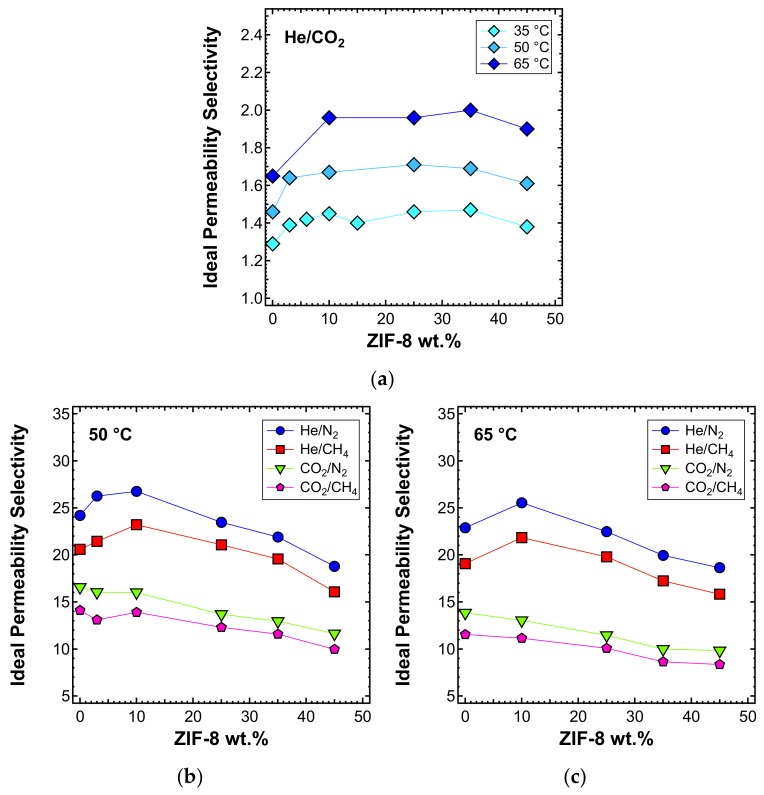
Ideal selectivity for (**a**) He/CO_2_ at 35, 50, and 65 °C and other gas couples at (**b**) 50 and (**c**) 65 °C in ZIF-8/PPO MMMs, with an upstream pressure of 1.3 bar.

The ideal selectivity was also estimated at 50 and 65 °C. In Figure 13a, the He/CO_2_ selectivity versus filler loading at the three different temperatures inspected is presented. A consistent increase of selectivity with temperature was observed. The shape of the curve remains similar at all temperatures, with an initial higher increase of selectivity for loadings below 10 wt%, followed by a stable trend and then a slight decrease at a filler loading of 45 wt%, for the reasons mentioned above. He/CO_2_ is the only gas pair, along with H_2_/CO_2_, for which the selectivity is enhanced by temperature [89]. This is because of the different nature of the two gases. Helium is a very small and non-condensable gas (T_c_ = 5.2 K), for which the permeability is controlled by diffusivity. CO_2_ is bigger and much more condensable (T_c_ = 304.2 K) and its permeability has a higher solubility contribution. Temperature enhances diffusivity, which is a kinetic property, but compromises solubility, which is a thermodynamic contribution. Overall, this leads to an increased He/CO_2_ selectivity. Given that a high temperature promotes the He/CO_2_ separation performance, these MMMs have potential applications at high temperatures. To test the thermal stability, TGA experiments were performed on the MMMs and the results are reported in Figure S1 of the Supplementary Information.

The selectivity of the MMMs inspected with respect to other gas couples is reported in Figure 13b and c for the temperatures of 50 and 65 °C, respectively. The optimal selectivity is obtained for a filler loading of 10% at both temperatures, for the gas He/CH_4_ and He/N_2_, respectively. On the other hand, the CO_2_/N_2_ and CO_2_/CH_4_ selectivity decreases with the filler content at both temperatures.

#### 3.5.4. Effect of Temperature

**Figure 14:**
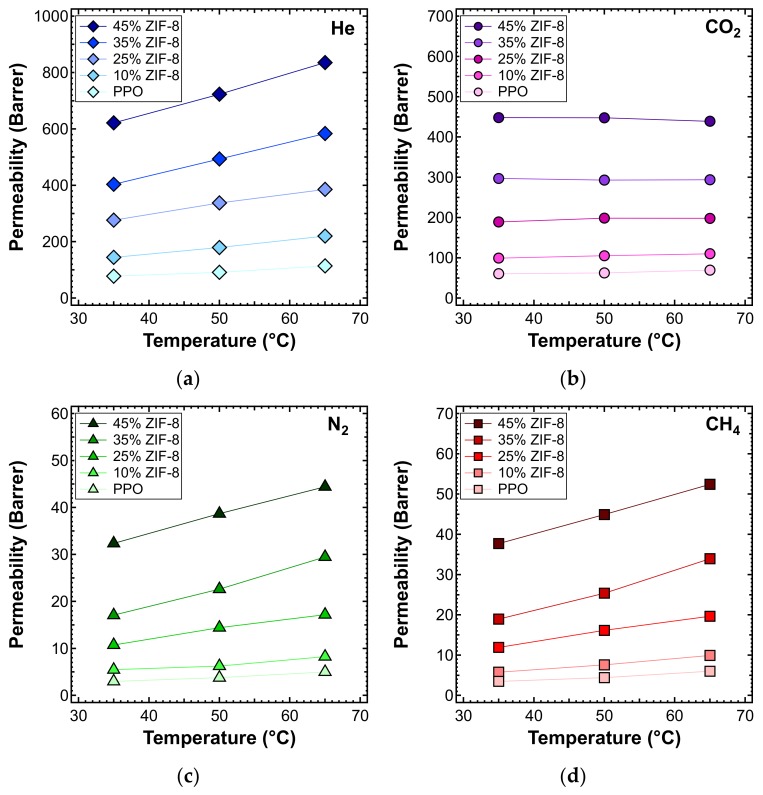
Permeability of (**a**) He, (**b**) CO_2_, (**c**) N_2_, and (**d**) CH_4_ at different temperatures, in the ZIF-8/PPO MMMs of different weight fractions of ZIF-8, from 0% to 45%.

**Figure 15:**
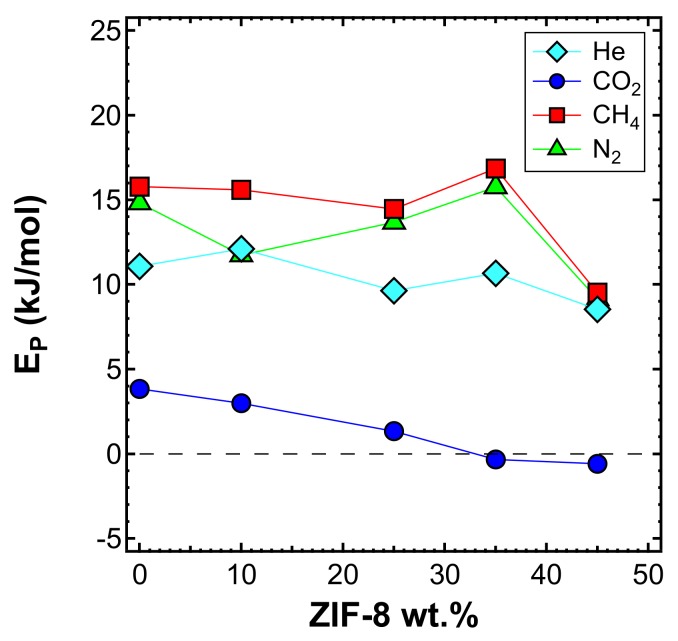
Activation energy of permeability as measured in the interval 35–65 °C for four gases in the various MMMs inspected, as a function of filler loading.

The effect of temperature on the transport properties was also investigated by plotting permeability data as a function of temperature for various filler loadings. This is reported in Figure 14 for each gas. These data can be further elaborated to obtain a more quantitative indication of the effect of temperature on permeability, namely, the energy contributions associated with the permeation process. The values calculated based on the data measured at three different temperatures are reported in Figure 15 and Table 5.

It can be noticed that the addition of ZIF-8 particles made the permeability a weaker function of the temperature. The plot clearly indicates that CO_2_ has a slight dependence on temperature (i.e., smaller values of $E_{P}$*),* which becomes negligible at high filler loadings. At 35 wt% and 45 wt%, $E_{P}$ had negative values, which means that the permeability decreases with an increasing temperature. This further enhances the He/CO_2_ selectivity, since the helium permeability has a larger dependence on temperature and remarkably increases with it. The main reason why CO_2_ permeability behaves in this way is because ZIF-8 provides a large sorption contribution at high loadings, and the loss of sorption outweighs the increase of diffusion while the temperature increases. N_2_ and CH_4_ show higher values for $E_{P}$, with CH_4_ consistently being the highest. All values drop in the case of the membrane containing 45 wt% of ZIF-8, consistent with the possible presence of voids, as previously discussed.

### 3.6. Diffusivity

#### 3.6.1. Effect of Filler Loading

**Figure 16:**
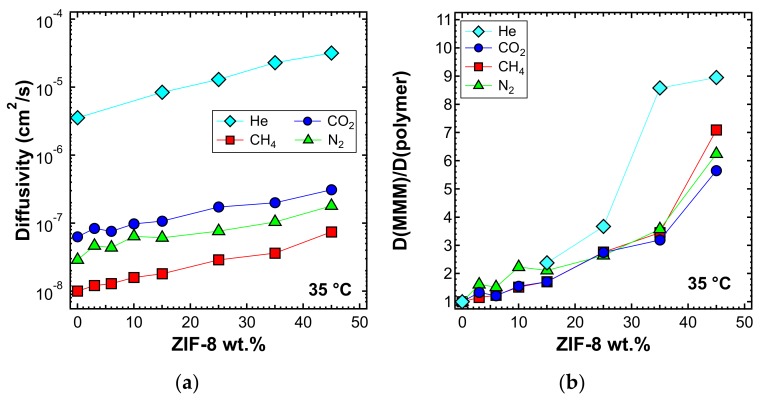
(**a**) Diffusivity and (**b**) relative diffusivity increase of various gases at 35 °C in ZIF-8/PPO mixed-matrix membranes (MMMs), estimated with the time-lag method.

The diffusivity of the different gases in the various MMMs was estimated from the permeation output through the time-lag method represented by Equation (4). Figure 16a shows the diffusivity at 35 °C as a function of ZIF-8 loading. Diffusivity values were in agreement with the following order: D(He) > D(CO_2_) > D(N_2_) > D(CH_4_), which is consistent with the values of the effective diameter of all the gas molecules investigated. In Figure 16b, the diffusivity ratio between each mixed-matrix membrane and the polymeric phase is reported. The diffusivity ratio was generally higher than unity for all gases and filler loadings. This indicates that the addition of filler enhanced the diffusion coefficient of gases, which possibly takes advantage of the fastest filler diffusive paths offered by the ZIF-8. Helium showed an inconsistent trend for MMMs at 3%, 6%, and 10% ZIF-8 loading, but we attribute this to the fact that the time-lag was often shorter than 2–3 s, which made it difficult to conduct accurate estimations in some cases. Overall, the trend remains clear. The enhancement of diffusivity induced by the filler addition is not as high as the one recorded for permeability: such a phenomenon indicates that the filler also promotes the gas solubility (Figure S5). A detailed analysis of the sorption properties is currently being conducted and will be the object of a forthcoming study.

**Figure 17:**
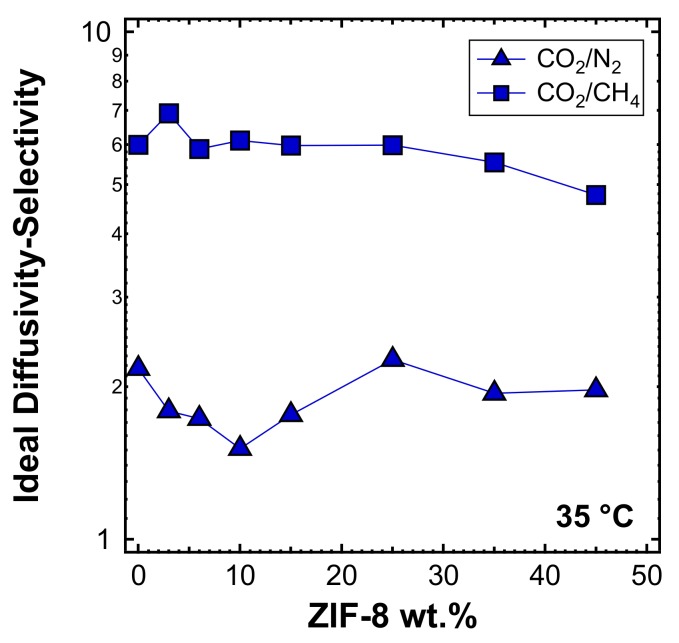
Ideal diffusivity-selectivity for CO_2_/N_2_ and CO_2_/CH_4_ at 35 °C in ZIF-8/PPO MMMs.

In Figure 17, the estimated values of diffusivity-selectivity for the couples CO_2_/N_2_ and CO_2_/CH_4_ are reported. In both cases, it can be seen that the diffusivity-selectivity is lower than the overall selectivity (Figure 10b), due to the fact that solubility plays a synergic role in separations involving CO_2_. The slightly decreasing trend of diffusivity-selectivity with the filler loading for both CO_2_/CH_4_ and CO_2_/N_2_ separations is consistent with the more preferable diffusion path offered by ZIF-8 in the MMMs.

**Figure 18:**
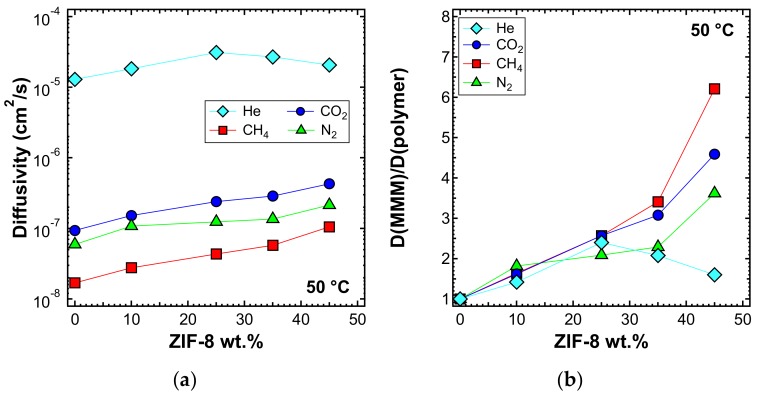
(**a**) Diffusivity and (**b**) relative diffusivity increase of various gases at 50 °C in ZIF-8/PPO MMMs, estimated with the time-lag method.

**Figure 19:**
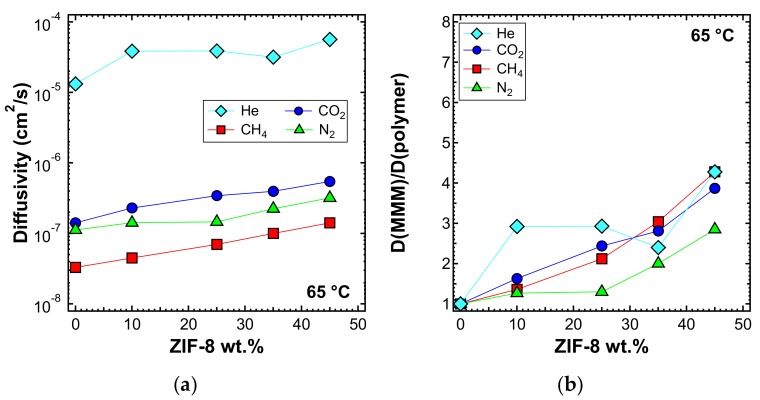
(**a**) Diffusivity and (**b**) relative diffusivity increase of various gases at 65 °C in ZIF-8/PPO MMMs, estimated with the time-lag method.

The diffusivity was also estimated with the time-lag method at higher temperatures, namely 50 and 65 °C, and is reported in Figure 18a and Figure 19a, respectively. In Figure 18b and Figure 19b, the values of the diffusivity ratio between the MMMs and the pure polymer at 50 and 65 °C, respectively, are reported. It can be noticed, from a qualitative point of view, that the diffusivity followed a similar behavior at all temperatures. However, the enhancement of diffusivity induced by the filler seems less remarkable at the higher temperatures. This could be explained by the lower activation energy of diffusion required for the MMMs than for PPO, which is indeed an aspect that will be analyzed quantitatively in the next section.

#### 3.6.2. Effect of Temperature

**Figure 20:**
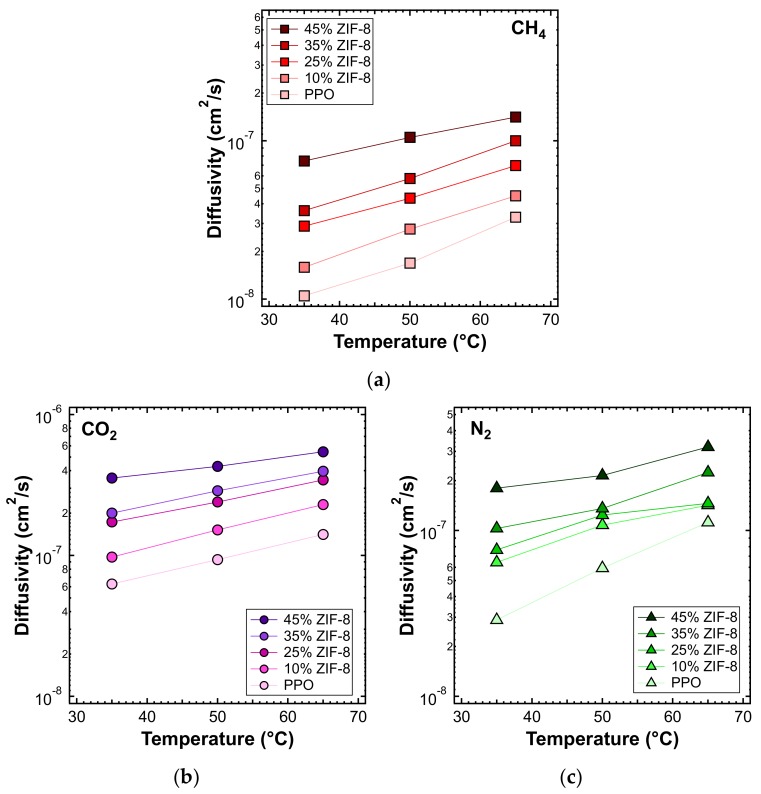
Diffusivity of (**a**) CH_4_, (**b**) CO_2_, and (**c**) N_2_ at different temperatures, in the ZIF-8/PPO MMMs of different weight fractions of ZIF-8, from 0% to 45%.

**Figure 21:**
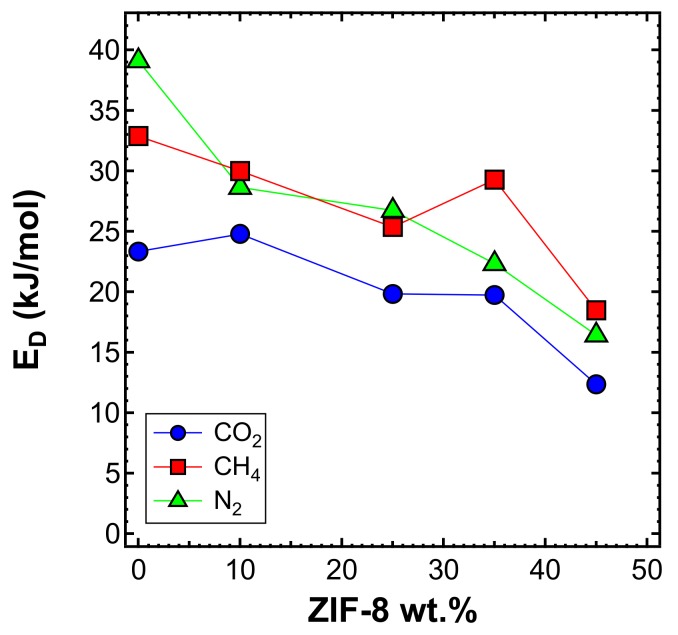
Activation energy of diffusion measured in the interval 35–65 °C for CO_2_, CH_4_, and N_2_ in the MMMs as a function of filler loading.

Figure 20 shows the diffusivity as a function of temperature, for the MMMs inspected and all gases, except helium. This is because higher temperatures result in diffusion occurring more quickly than at 35 °C, making the time-lag even shorter and less accurate. On the other hand, the diffusivity of the other gases, with a few exceptions, followed Arrhenius law in all the mixed matrices considered. Therefore, we were able to calculate the activation energy of diffusion, which is reported in Figure 21 and Table 6. The values of activation energy for diffusion were higher than the respective values of $E_{P}$, since the sorption process is always exothermic and involves negative values of sorption enthalpy ($\Delta H_{S}$). Furthermore, as in the case of $E_{P}$, the values decreased with an increasing ZIF content. This aspect indicates that the addition of filler to the polymer lowered the energetic barrier of the diffusion process, possibly due to the presence of filler pores available for diffusion.

### 3.7. Estimated Solubility

**Figure 22:**
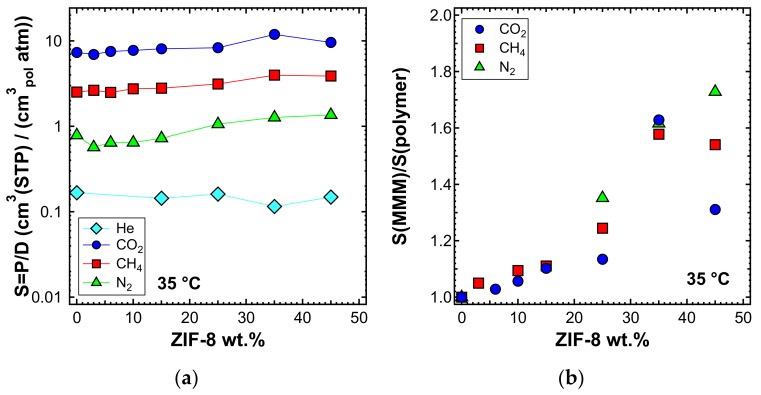
Estimated solubility (**a**) and solubility ratio (**b**) at 35 °C in the mixed-matrix membranes inspected. Values evaluated as P/D.

The solubility coefficient was calculated as the ratio between permeability and diffusivity, assuming the validity of the solution-diffusion model. The values are shown in Figure 22a for the experiment performed at 35 °C. A slight increase of the solubility coefficient at high filler loadings was recorded. Figure 22b shows that CO_2_, N_2_, and CH_4_ experienced a similar enhancement of solubility, while for He, there was no such effect. The data indicate that the filler enhanced the gas permeability of the polymer by mainly acting on the diffusivity but also, to a non-negligible extent, on the ability of the membrane to absorb gas.

#### Effect of Temperature: Sorption Enthalpy ($\Delta H_{S}$)

**Figure 23:**
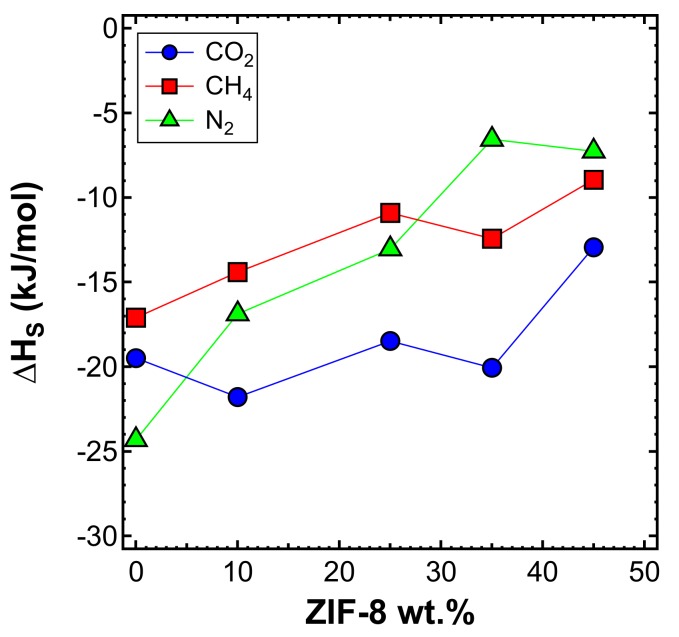
Heat of sorption estimated in the interval 35–65°C for three gases in the various mixed-matrix membranes inspected, as a function of filler loading.

The sorption enthalpy can be estimated by subtracting the activation energy of diffusion from that of permeation according to Equation (7). Figure 23 shows the trend of $\Delta H_{S}$ with the ZIF-8 content, and Table 7 reports its values. CO_2_ showed, on average, the most negative values among all gases, which means that CO_2_ sorption is more favorable than that of CH_4_ and N_2_. This is related to the higher condensability of CO_2_, as expected due to being the most soluble gas, as observed in Figure 22a. The absolute value of the sorption enthalpy decreases with an increasing filler content.

### 3.8. Performance Evaluation Using a Robeson Upper Bound Plot

**Figure 24:**
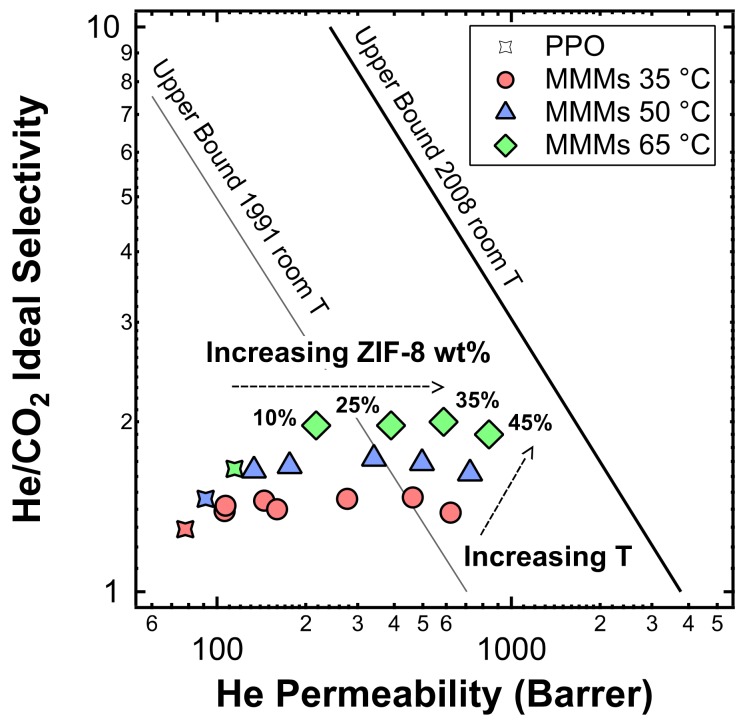
Positioning of the MMMs studied in this work in a Robeson plot for He/CO_2_ separation. For data collected at 35 °C, filler loadings of 0%, 3%, 6%, 10%, 15%, 25%, 35%, and 45% in weight of ZIF-8 were tested. For data collected at 50 °C, filler loadings are equal to 0wt%, 3 wt%, 10 wt%, 25 wt%, 35 wt%, and 45 wt%, while for data collected at 65 °C, only data at wt %, 10 wt%, 25 wt%, 35 wt%, and 45 wt% were collected. The effect of the temperature is also shown.

The performances of the composite membranes developed in this work were plotted on a Robeson plot for the He/CO_2_ pair, featuring both the 1991 [6] and 2008 [7] upper bounds. The results are reported in Figure 24. The ZIF-8/PPO MMMs at 35 wt% and 45 wt% overcame the 1991 upper bound for the test at room temperature, while at 65 °C, the film at 25 wt% also surpassed the limit. These results were achieved because MMMs were simultaneously more permeable and more selective when compared to PPO. It must be noted, however, that the Robeson plots were obtained based on room temperature data for this gas pair. Robeson plots for He/N_2_, He/CH_4_, CO_2_/N_2_, and CO_2_/CH_4_ gas pairs are reported in Figure S4 of the Supplementary Information.

### 3.9. Comparison with Other MMMs

**Figure 25:**
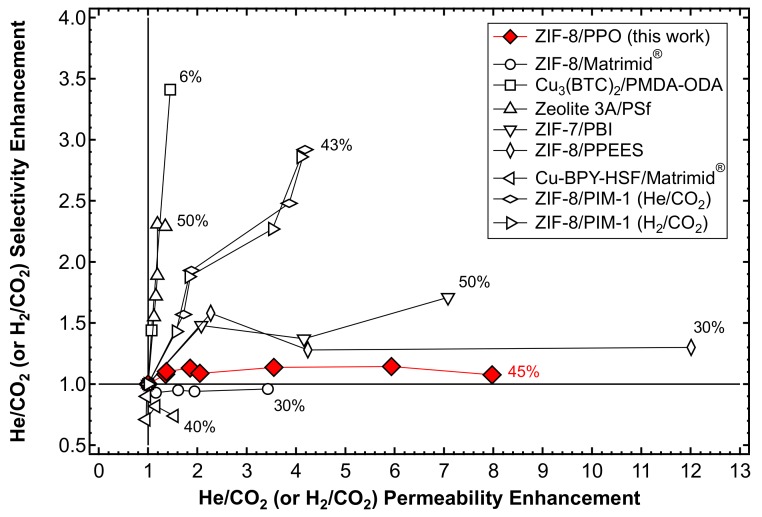
Effect of the addition of size-selective fillers on the He (or H_2_) permeability and He(or H_2_)/CO_2_ selectivity of a series of glassy polymers with respect to the pure polymer matrix. The maximum loading achieved by each MMM is represented next to each series of data. Results from references [48,54,56,60,61,90,91].

In the present section, the results are compared in terms of the relative permeability and selectivity enhancement with respect to He/CO_2_ or H_2_/CO_2_ separation, obtained by adding H_2_-selective fillers to glassy polymers. The data shown in Figure 25 refer to MMMs formed by ZIF-7/Polybenzimidazole (PBI) [56]; Zeolite 3A/Polysulfone [54]; ZIF-8 blended with different polymer matrices such as Matrimid^®^ [48], PIM-1 [61], and PPEES [60]; Cu-BPY-HSF/Matrimid^®^ [90]; and Cu_3_(BTC)_2_/PMDA-ODA [91]. ZIF-8/PIM-1 MMMs showed a significant and balanced increase in the selectivity and permeability. In particular, He and H_2_ permeabilities were improved by a factor of ~4, while He/CO_2_ and H_2_/CO_2_ selectivity exhibited a three-fold increase. However, PIM-1 is intrinsically different from PPO in that it is CO_2_-selective over H_2_ in its pure unfilled state, as well as Matrimid^®^. The permeability enhancement observed by adding ZIF-8 to PPO, as assessed in this work, is among the highest ever recorded, second to only the 30 wt% PPEES/ZIF-8 membrane produced by Díaz et al. [60]. The addition of the hydrophilic Zeolite 3A to PSf, characterized by a smaller pore size, on the other hand, appreciably enhances the selectivity, but only slightly increases the permeability [54]. This effect is most likely due to the fact that Zeolite 3A has a higher selectivity than ZIF-8 given the rigidity of the inorganic cage. It can be said that the behavior of the mixed-matrix membranes inspected in this work resembles that of other materials obtained by mixing the same filler, ZIF-8, with a polymer that features similar initial properties in terms of the permeability and selectivity with respect to PPO. The data indeed indicate that the addition of ZIF-8 to a size-selective polymer can remarkably enhance the permeability, but has a limited effect on the selectivity, due to the intrinsically moderate selectivity of ZIF-8.

Finally, it can be noticed that the behavior of the mixed-matrix membranes obtained here is, at least qualitatively, intermediate between that of the pure polymer and that of the pure filler. This is strictly related to the optimization of a casting protocol that allows filler dispersion in solution and defect-free film formation. Such a result is in agreement with the fact that, according to SEM analysis and density tests, the composite materials show good adhesion between the polymer and the metalorganic phases, indicating that the properties of the composite do not significantly deviate from ideality. This can be considered, by all means, a qualitative structure-property correlation which can guide the design of novel mixed-matrix materials with targeted properties for specific separation applications by appropriate combinations of polymers and fillers with known initial properties.

## 4. Conclusions

In this work, we fabricated mixed-matrix membranes based on PPO and variable amounts of ZIF-8, with the goal of enhancing the sieving ability to achieve better H_2_/CO_2_ separation. A preparation protocol that allowed us to obtain membranes with loadings of filler as high as 45 wt% was defined. Morphological analysis highlighted the presence of generally well-dispersed filler particles with generally good interface adhesion between the polymer and filler, but also the formation of some aggregates at high loading. Thermal analysis showed the absence of residual solvents or moisture, the amorphous structure of the membranes, and the linear increase of the glass transition temperature with an increasing filler content in the polymer matrix. The buoyancy tests performed allowed us to estimate that the density of the composite membranes follows an additive behavior, based on the pure polymer and the theoretical density of the ZIF-8 crystal, which indicates good adhesion of the polymer and filler phase and no formation of voids at the interface.

Permeability tests were performed on membranes containing various contents of ZIF-8 at temperatures between 35 and 65 °C. The addition of ZIF-8 to the polymer produced a monotonous increase of permeability among all four gases tested, with factors as high as 8. This trend was obeyed at all temperatures. He/CO_2_ selectivity, on the other hand, increased, to a smaller extent, up to a loading of ZIF-8 of about 35 wt%, due to the fact that the filler has a moderate selectivity with respect to this gas pair. The gas diffusivity also monotonously increased with the ZIF-8 content, for all gases and at each temperature. However, the enhancement of diffusivity alone does not justify the observed enhancement of permeability and, according to the solution diffusion-model, we were able to assess that there was also a beneficial effect of ZIF-8 addition on the gas solubility in the MMMs.

An analysis of the activation energies showed that the presence of filler in the polymer matrix made transport in the composite films easier. Indeed, $E_{P}$ and $E_{D}$ decreased with an increasing ZIF-8 content in the membrane. This behavior is compatible with the availability of a higher free volume for gas transport in the presence of filler particles.

The Robeson plot for He/CO_2_ separation indicates that the addition of ZIF-8 pushes the MMM performance above the room temperature 1991 upper bound, and close to the room temperature 2008 upper bound. The temperature increase also yielded a simultaneous increase of permeability and selectivity, indicating that such membranes can have potential for applications at high temperatures. However, further optimization is required, especially in terms of the selectivity, before proceeding to produce thin film membranes required for upscaling of the materials.

The present results, together with those obtained previously on materials characterized by properties similar to PPO, i.e., a moderate selectivity for the H_2_/CO_2_ and He/CO_2_ gas pairs, indicate that the addition of ZIF-8 to such materials leads to mixed-matrix membranes which are defect-free and can show remarkably higher permeabilities than initial values, thanks to the intrinsic filler permeability. However, the increase of selectivity achievable is not significant, due to the intrinsically moderate selectivity of ZIF-8 for such gas pairs. However, the properties of the mixed-matrix membranes inspected here are intermediate in relation to those of the pure polymer and the pure filler, as expected from defect-free composite materials: such a result can guide the design of novel composite materials with tunable separation properties by appropriate combinations of polymer and filler.

## Figures and Tables

**Table 1:** Bulk physical properties of amorphous poly(2,6-dimethyl-1,4-phenylene oxide) (PPO).

Polymer	ρ (25 °C)	$T_{g}$	%FFV [52]	Refractive Index [52]	Average Molecular Weight [53]
**PPO**	g/cm^3^	°C			g/mol
** 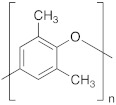 **	1.06	213	19	1.573	59,000

**Table 2:** Physical properties, composition, and reticular structure of ZIF-8.

Filler	Composition [49]	Net	d_a_ [34]	d_p_ [34]	Surface Area (BET) [49]	Theoretical Density	Thermal Stability [49]	Hydrophilicity [49,64]
			Å	Å	m^2^/g	g/cm^3^	°C	
**ZIF-8**	Zn(MeIM)_2_	sod	3.4	11.6	1630	0.95 [55] 0.93 [61]	550	Hydrophobic

**Table 3:** The single-gas permeability and ideal selectivity of pure ZIF-8 membranes [55].

**Thickness** (μm)	~30	~20	~20
Ref.	[40]	[41]	[42]
**Permeance** (10^−8^ mol m^−2^ s^−1^ Pa^−1^)			
H_2_	6.04	17.3	8.23
N_2_	0.52	1.49	0.69
CH_4_	0.48	1.33	0.63
CO_2_	1.33	4.45	/
**Permeability**			
(Barrer)			
H_2_	5411	10,333	4916
N_2_	466	890	412
CH_4_	430	794	376
CO_2_	1192	2658	/
**Ideal Selectivity**			
H_2_/CO_2_	4.54	3.89	/
CO_2_/N_2_	2.56	2.99	/
CO_2_/CH_4_	2.77	3.35	/
H_2_/CH_4_	12.6	13.0	13.1
H_2_/N_2_	11.6	11.6	11.9

**Table 4:** Pure gas permeability and ideal selectivity in PPO and ZIF-8/PPO MMMs. Tests were performed at 35 °C and an upstream pressure of 1.3 bar.

ZIF-8 Loading (wt%)	Pure Gas Permeability (Barrer^a^)				Ideal Selectivity				
	He	N_2_	CH_4_	CO_2_	He/CO_2_	CO_2_/N_2_	CO_2_/CH_4_	He/CH_4_	He/N_2_
0 (PPO) [88]	77.9 ± 2.3	2.99 ± 0.07	3.47 ± 0.09	60.6 ± 1.5	1.29	20.2	17.4	22.3	26.0
3	105.8 ± 2.5	3.49 ± 0.08	4.20 ± 0.10	76.1 ± 1.9	1.39	21.8	18.1	25.2	30.3
6	106.7 ± 2.4	3.71 ± 0.08	4.25 ± 0.10	75.3 ± 1.7	1.42	20.3	17.7	25.4	28.8
10	144.3 ± 3.6	5.67 ± 0.13	5.76 ± 0.14	99.5 ± 2.4	1.45	17.5	17.3	24.9	25.4
15	159.7 ± 1.5	5.83 ± 0.06	6.61 ± 0.07	114.1 ± 1.1	1.40	19.6	17.3	24.2	27.4
25	276.4 ± 10.4	10.7 ± 0.4	11.9 ± 0.5	189.0 ± 7.2	1.46	17.7	15.9	23.2	25.9
35	462.0 ± 31.0	17.2 ± 1.1	18.9 ± 1.3	314.2 ± 21.0	1.47	18.2	16.6	24.4	26.8
45	620.9 ± 54.0	32.3 ± 2.8	37.9 ± 3.3	448.7 ± 38.9	1.38	13.9	11.8	16.4	19.2

**Table 5:** Activation energy of permeability in the range of 35–65 °C for ZIF-8/PPO MMMs at different filler loadings.

ZIF-8 Loading (wt%)	$E_{P}\;(\mathrm{kJ}/\mathrm{mol})$			
	He	N_2_	CH_4_	CO_2_
0 [50]	9.7	9.8	12.1	1.5
0	11.07	14.81	15.78	3.84
10	12.11	11.74	15.59	2.99
25	9.64	13.68	14.46	1.34
35	10.66	15.76	16.85	0.33
45	8.53	9.15	9.52	0.59

**Table 6:** Activation energy of diffusion in the range of 35–65 °C for ZIF-8/PPO MMMs at different filler loadings.

ZIF-8 Loading (wt%)	E_D_ (kJ/mol)		
	N_2_	CH_4_	CO_2_
**0** [50]	22.4	29.4	23.4
**0**	39.10	32.87	23.33
**10**	28.62 *	29.99	24.78
**25**	26.73 *	25.37	19.82
**35**	22.32	29.28	19.73
**45**	16.42	18.47	12.35

* Activation energy of diffusion was calculated using diffusivity data at 35 and 50 °C.

**Table 7:** Heat of sorption ∆H_S_ in the range of 35–65 °C for ZIF-8/PPO MMMs at different filler loadings.

ZIF-8 Loading (wt%)	∆HS (kJ/mol)		
	N2	CH4	CO_2_
**0** [50]	−12.6	−17.3	−21.9
**0**	−24.32	−17.10	−19.49
**10**	−16.88 *	−14.40	−21.79
**25**	−13.05 *	−10.91	−18.48
**35**	−6.56	−12.43	−20.06
**45**	−7.27	−8.95	−12.94

* Activation energy of diffusion was calculated using the activation energy of diffusion calculated using data at 35 and 50 °C.

## Supplementary Materials

The following are available online at https://www.mdpi.com/2077-0375/10/4/56/s1: Figure S1: TGA analysis results of PPO (red), ZIF-8 (black), 15 wt% ZIF-8/PPO (blue), and 25 wt% ZIF-8/PPO (green). Tests were performed in nitrogen atmosphere. PPO and MMMs were pre-treated at 200 °C overnight and then normally exposed to air for days/weeks, while ZIF-8 powder was tested as received; Figure S2: First DSC scan of a sample of PPO casted by inducing slow solvent evaporation, with subsequent formation of crystal domains and rupture of the membrane. Before performing DSC, the film was not pre-treated; Figure S3: SEM images of the cross-section of PPO/ZIF-8 mixed-matrix membranes at different loadings and magnitudes: (a) 25 wt.%, (b) 25 wt.%, (c) 25 wt.%, (d) 10 wt.%, (e) 6 wt.%, (f) 45 wt.%, (g) 25 wt.%, (h) 10 wt.%, (i) 25 wt.%, and (j) 25 wt.%; Figure S4: Positioning of the MMMs studied in this work in Robeson plots for (a) He/N_2_, (b) He/CH_4_, (c) CO_2_/N_2_, and (d) CO_2_/CH_4_ separations. For data collected at 35 °C, filler loadings of 0%, 3%, 6%, 10%, 15%, 25%, 35%, and 45% in weight of ZIF-8 were tested. For data collected at 50 °C, filler loadings were equal to 0 wt%, 3 wt%, 10 wt%, 25 wt%, 35 wt%, and 45 wt%, while for data collected at 65 °C, only data at 0 wt%, 10 wt%, 25 wt%, 35 wt%, and 45 wt% were collected. The effect of the temperature is also shown; Figure S5: Parity plot of diffusivity enhancement and permeability enhancement due to the addition of ZIF-8 to PPO at 35 °C for (a) He, (b) CO_2_, (c) N_2_, and (d) CH_4_; Table S1: Pure gas permeability and ideal selectivity in PPO and ZIF-8/PPO MMMs. Tests were performed at 50 °C and 1.3 bar was employed as the upstream pressure; Table S2: Pure gas permeability and ideal selectivity in PPO and ZIF-8/PPO MMMs. Tests were performed at 65 °C and 1.3 bar was employed as the upstream pressure.
