# Supplementary material for: Enhancing the Separation Performance of Glassy PPO with the Addition of a Molecular Sieve (ZIF-8): Gas Transport at Various Temperatures
Source: Membranes (Basel). 2020 Mar 27;10(4):56. doi: 10.3390/membranes10040056 (PMC7231394; doi:10.3390/membranes10040056)
Supplement: Supplementary file 1 [file membranes-10-00056-s001.pdf]

membranes

Supplementary Material

Enhancing the separation performance of glassy PPO with the addition of a molecular sieve (ZIF-8): gas transport at various temperatures

Francesco M. Benedetti [1,2,*], Maria Grazia De Angelis [1,*] Micaela Degli Esposti [1], Paola Fabbri [1], Alice Masili [3], Alessandro Orsini,[3] Alberto Pettinau [3]

[1] Department of Civil, Chemical, Environmental and Materials Engineering, University of Bologna, Italy grazia.deangelis@unibo.it

[2] Present address: Department of Chemical Engineering, Massachusetts Institute of Technology, Cambridge, Massachusetts USA; fmben@mit.edu

[3] Sotacarbo S.p.A., Grande Miniera di Serbariu, Carbonia (CA), Italy, alessandro.orsini@sotacarbo.it

S1. TGA results

Tests were performed in nitrogen atmosphere. PPO and MMMs were pre-treated at 200 °C overnight and then normally exposed to air for days/weeks, while ZIF-8 powder was tested as received. Thermogravimetric analysis was already performed on ZIF-8 by other authors. Park *et al.*[1] reported a weight loss of 28.3% once the temperature reached 450 °C. This was addressed by the authors as an escape of the residual molecules from the as-synthesized MOF (*e.g.* DMF, H_2O etc.). However, the commercial ZIF-8 used in this work showed almost no weight loss up to the same temperature (**Figure S1**), likely because it comes already purified. However, the two materials showed the same behavior from 600 °C to 800 °C. Zhang *et al.*[2] tested the same ZIF-8 we used in this work below its decomposition temperature, previously saturated in water vapor. Water-saturated ZIF-8 lost only a 3% of its weight, as a proof of its hydrophobic behavior and great thermal stability. Along with the MOF, PPO revealed a surprisingly high thermal resistance up to 400 °C as shown in **Figure S1**, corroborating that ZIF-8/PPO MMMs are suitable for high temperature applications.

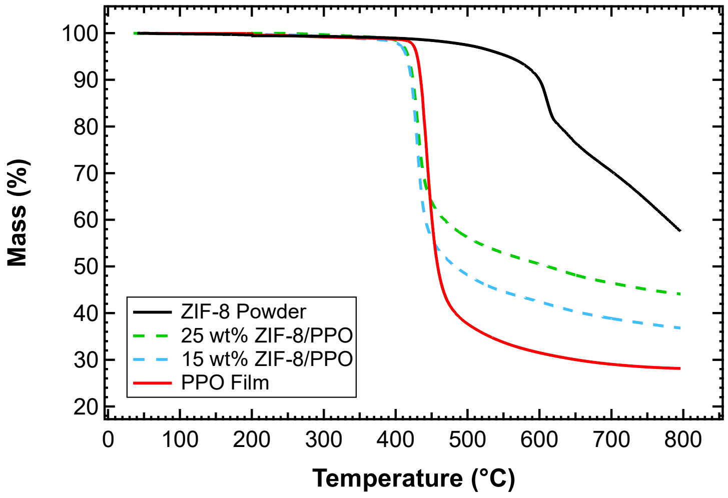

Figure S1: TGA analysis results of PPO (red), ZIF-8 (black), 15 wt% ZIF-8/PPO (blue) and 25 wt% ZIF-8/PPO (green).

S2. DSC on semi-crystalline PPO

It must be noticed that slower casting at room temperature (*i.e.*, complete evaporation of $CHCl_3$ in 3 days), resulted in the formation of semi-crystalline PPO samples, as verified via DSC analysis, reported in **Figure S2a** and rupture of the membrane during film formation (**Figure S2b**). The polymer was not transparent, conversely to the amorphous one, but white, which is consistent with the formation of crystal domains. Before performing DSC the film was not pre-treated.

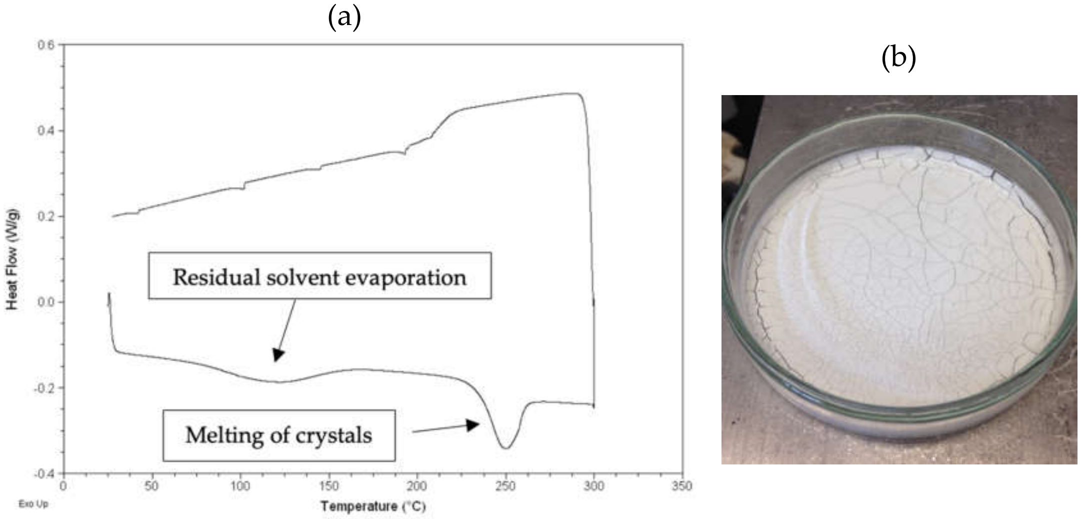

Figure S2: First DSC scan of a sample of PPO casted inducing slow solvent evaporation, with formation of crystal domains and rupture of the membrane.

S3. Supplementary SEM images

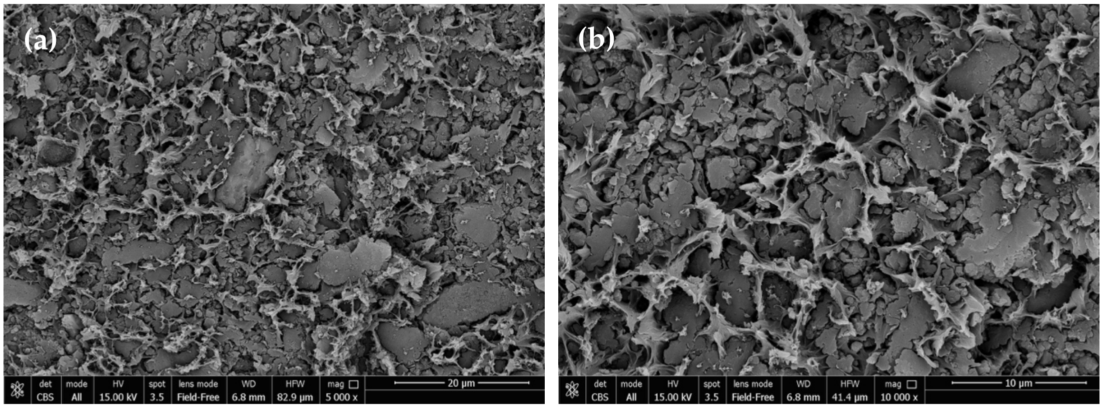

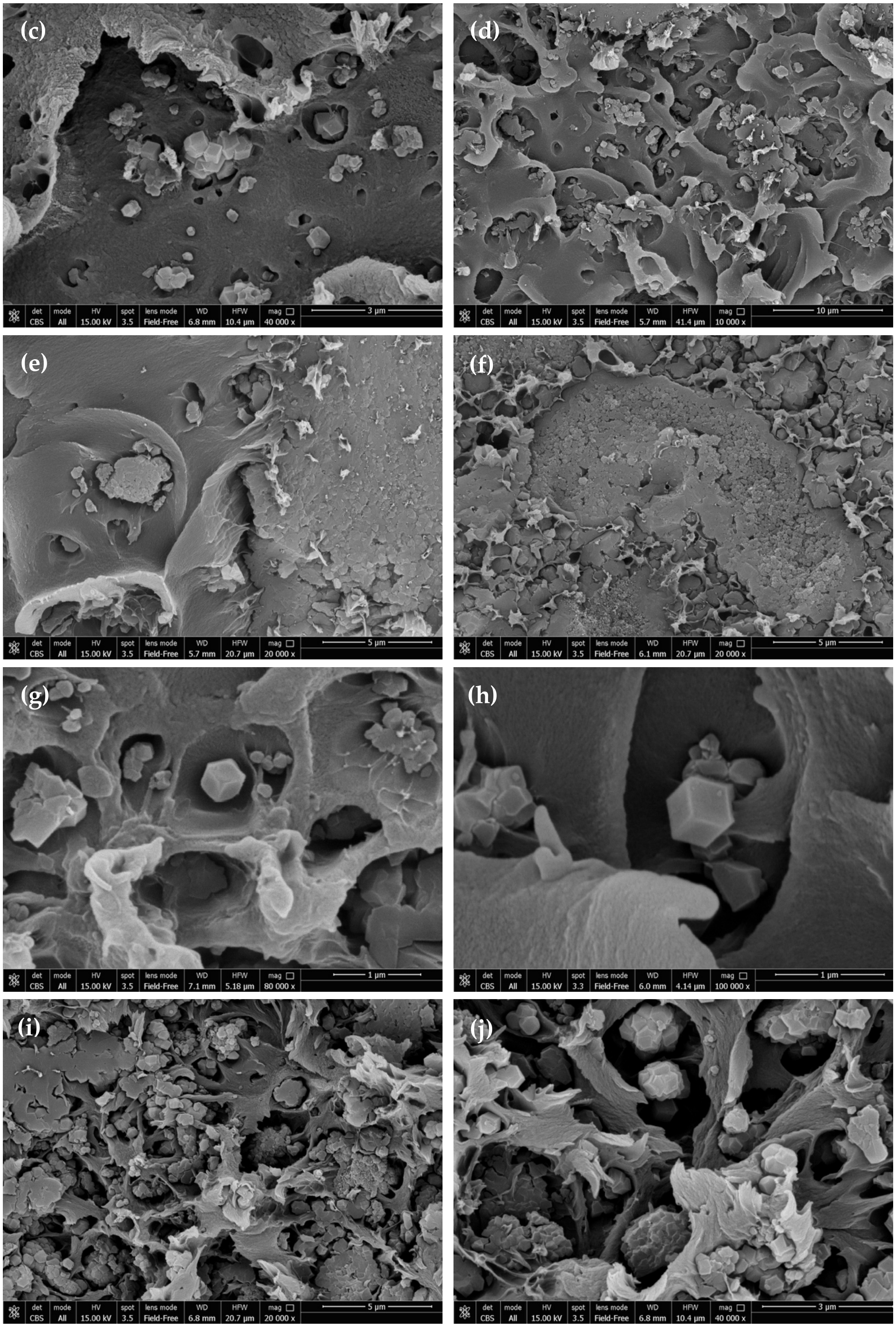

Figure S3: SEM images of the cross-section of PPO/ZIF-8 mixed matrix membranes at different loadings and magnitudes: (a) 25 wt.%, (b) 25 wt.%, (c) 25 wt.%, (d) 10 wt.%, (e) 6 wt.%, (f) 45 wt.%, (g) 25 wt.%, (h) 10 wt.%, (i) 25 wt.% and (j) 25 wt.%.

S4. Supplementary permeability results at high temperature

Table S1: Pure gas permeability and ideal selectivity in PPO and ZIF-8/PPO MMMs. Tests were performed at 50 °C and 1.3 bar as the upstream pressure.

ZIF-8 loading (wt.%)	Pure gas permeability (Barrer[a])				Ideal selectivity				
	He	N_2	CH_4	CO_2	He/CO_2	CO_2/N_2	CO_2/CH_4	He/CH_4	He/N_2
**0 (PPO)**	91.3	3.77	4.43	62.5	1.46	16.6	14.1	20.6	24.2
**3**	133.1	5.07	6.21	81.3	1.64	16.0	13.1	21.4	26.3
**10**	176.1	6.23	7.59	105.1	1.67	16.8	13.7	23.2	28.2
**25**	340.6	14.4	16.1	198.5	1.72	13.8	12.3	21.1	23.6
**35**	496.3	22.6	25.4	293.7	1.69	13.0	11.6	19.6	21.9
**45**	722.9	38.7	44.9	447.5	1.62	11.6	9.96	16.1	18.7

[a]Barrer: 10^{-10} cm^3 cm cm^{-2} s^{-1} $(cmHg)^{-1}$

Table S2: Pure gas permeability and ideal selectivity in PPO and ZIF-8/PPO MMMs. Tests were performed at 65 °C and 1.3 bar as the upstream pressure.

ZIF-8 loading (wt.%)	Pure gas permeability (Barrer[a])				Ideal selectivity				
	He	N_2	CH_4	CO_2	He/CO_2	CO_2/N_2	CO_2/CH_4	He/CH_4	He/N_2
**0 (PPO)**	114.4	5.00	6.00	69.3	1.65	13.85	11.54	19.07	13.85
**10**	216.5	8.48	9.91	110.1	1.97	12.99	11.11	21.84	12.99
**25**	389.6	17.2	19.7	198.0	1.97	11.52	10.05	19.79	11.52
**35**	587.1	29.5	34.1	294.2	2.00	9.98	8.64	17.24	9.98
**45**	837.4	44.9	52.9	441.5	1.90	9.83	8.35	15.83	9.83

S5. Supplementary Robeson upper bound plots

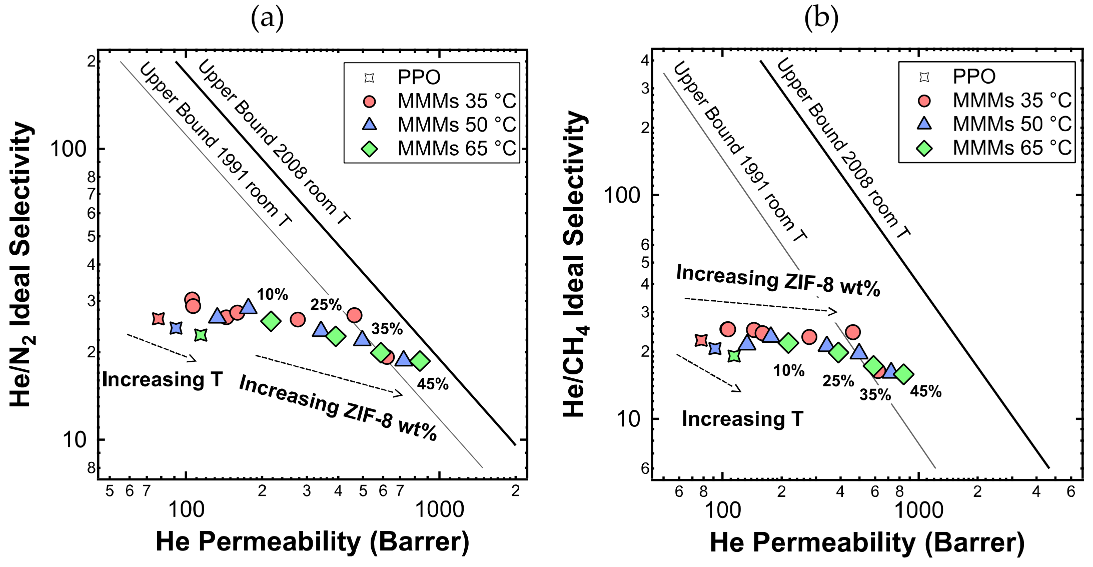

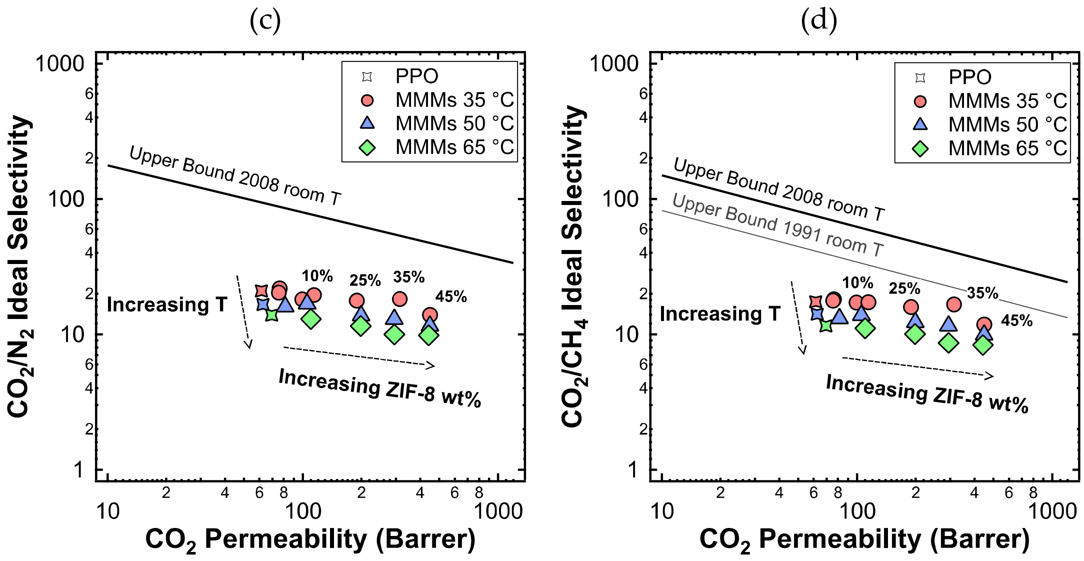

Figure S4: Positioning of the MMMs studied in this work in Robeson plots for (a) He/N_2, (b) He/CH_4, (c) CO_2/N_2 and (d) CO_2/CH_4 separations. For data collected at 35 °C, filler loadings of 0%, 3%, 6%, 10%, 15%, 25%, 35%, and 45% in weight of ZIF-8 were tested. For data collected at 50 °C, filler loadings are equal to 0 wt%, 3 wt%, 10 wt%, 25 wt%, 35 wt%, and 45 wt%, while for data collected at 65 °C just data at 0 wt%, 10 wt%, 25 wt%, 35 wt%, and 45 wt% were collected. The effect of the temperature is shown as well.[3,4]

S6. Permeability vs. diffusivity enhancement

In this section we compare the effect of filler addition on the permeability and the diffusivity. In particular, the idea is to see whether the permeability behavior is uniquely correlated to the diffusivity, or there is also an effect of the filler addition on the solubility, which will be reported in the part II of this work. To do so, in Error! Reference source not found. we have plotted against each other the permeability enhancement and the diffusivity enhancement induced by the filler in the MMMs analyzed at 35 °C for all the gases considered. In particular, it can be seen that for helium almost all the data fall exactly on the parity line, indicating that the filler enhances the permeability of helium essentially by acting on the diffusivity, and not the solubility. For all the other cases, the permeability enhancement is equal to the diffusivity enhancement at the low filler loadings up to 15 wt%, but becomes larger for larger filler loadings. This behavior indicates that, for such gases, the filler also enhances the solubility of gases in the polymer to a significant extent.

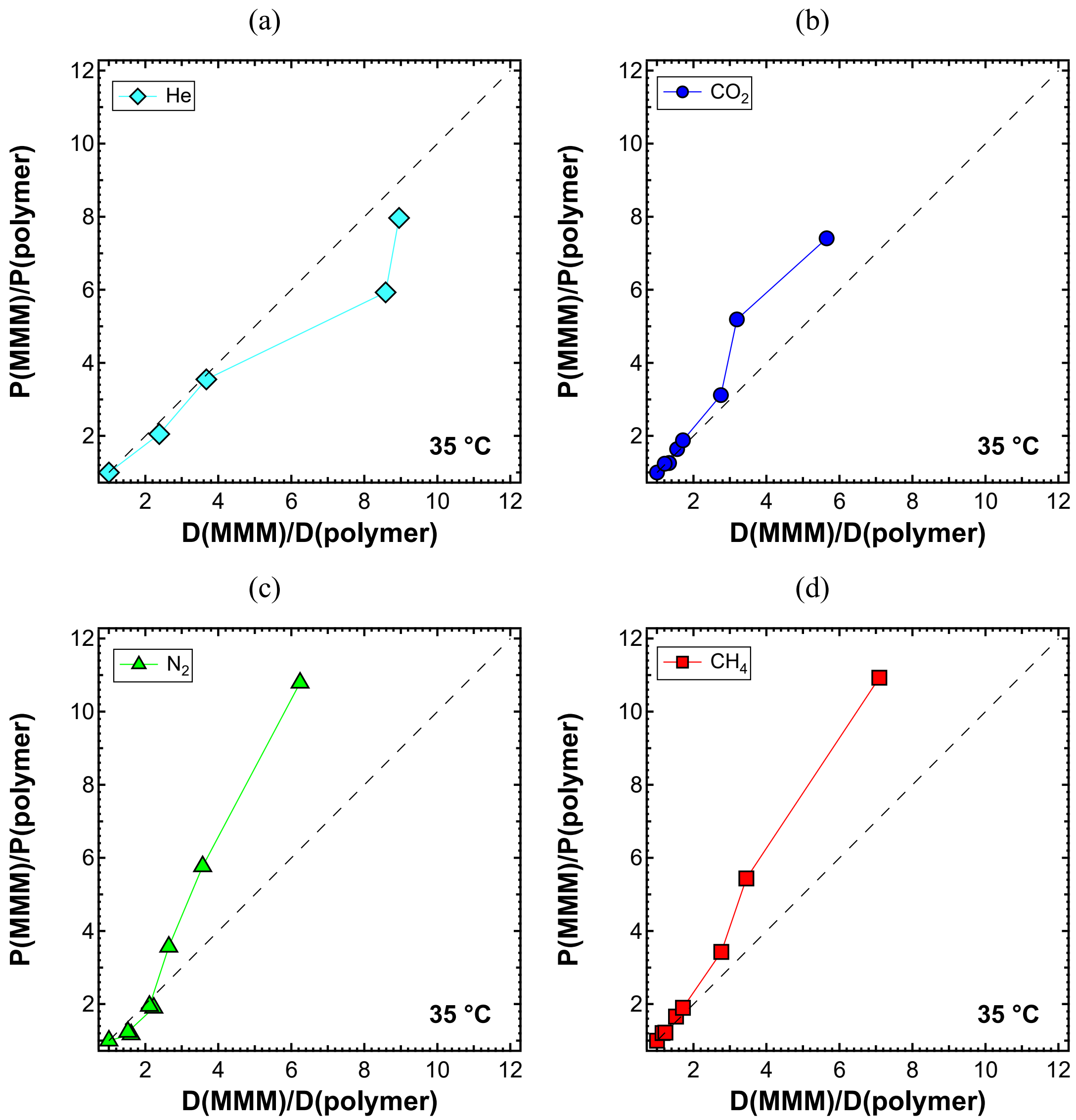

Figure S5: Parity plot between diffusivity enhancement and permeability enhancement due to addition of ZIF-8 to PPO at 35 °C for (a) He, (b) CO_2, (c) N_2, (d) CH_4.

References

1. K.S. Park, Z. Ni, A.P. Cote, J.Y. Choi, R. Huang, F.J. Uribe-Romo, H.K. Chae, M. O'Keeffe, O.M. Yaghi, Exceptional chemical and thermal stability of zeolitic imidazolate frameworks, Proc. Natl. Acad. Sci. 103 (2006) 10186–10191. doi:10.1073/pnas.0602439103.
2. C. Zhang, Y. Dai, J.R. Johnson, O. Karvan, W.J. Koros, High performance ZIF-8/6FDA-DAM mixed matrix membrane for propylene/propane separations, J. Memb. Sci. 389 (2012) 34–42. doi:10.1016/j.memsci.2011.10.003.
3. L.M. Robeson, Correlation of separation factor versus permeability for polymeric membranes, J. Memb. Sci. 62 (1991) 165–185. doi:10.1016/0376-7388(91)80060-J.
4. L.M. Robeson, The upper bound revisited, J. Memb. Sci. 320 (2008) 390–400. doi:10.1016/j.memsci.2008.04.030.

© 2020 by the authors. Submitted for possible open access publication under the terms and conditions of the Creative Commons Attribution (CC BY) license (http://creativecommons.org/licenses/by/4.0/).
